# Supplementary material for: ‘Power plays plus push’: experts’ insights into the development and implementation of active tuberculosis case-finding policies globally, a qualitative study
Source: BMJ Open. 2020 Jun 3;10(6):e036285. doi: 10.1136/bmjopen-2019-036285 (PMC7282330; doi:10.1136/bmjopen-2019-036285)
Supplement: Supplementary data [file bmjopen-2019-036285supp002.pdf]

Additional file 2. Interview guide

Basic information and active case-finding (ACF) policy and implementation work in your organization

1. What is your role in ACF policy development and/or implementation?
    - Since when have you been working in this role?
2. Could you briefly describe (current) ACF policy development and implementation supported by your organization?
    - What is the status of implementation and scale-up?
    - What do monitoring and evaluation efforts look like?
        i. Which indicators are being used?
        ii. Is there any data available/published?
    - What are the future plans for ACF in this context?

Personal view/values and preferences

3. What are the benefits of ACF in your view?
    - … at the level of the individual?
    - … at the community level?
    - … at the level of the health system?
    - … compared to other interventions for early case detection?
    - Have you/has your organization deprioritized other activities in order to prioritize resources for ACF?
        i. If so, why do you/does your organization prioritize ACF?
    - What other activities for early case detection are you/is your organization engaging in? (e.g. improve lab/diagnostic services, train health workers in identifying people with possible tuberculosis (TB), reduce financial/access barriers, address social protection)
    - Do you think the scale-up of ACF is essential for reaching the goals outlined in the End TB Strategy?
        i. If so, what's the most important action to achieve this scale-up?
        ii. Should anything else instead be prioritized less?
4. What are the risks of ACF in your opinion? (e.g. harm for individual, stigma and discrimination, cost, operational risks)
    - … at the level of the individual?
    - … at the community level?
    - … at the level of the health system?
    - … compared to other interventions for early case detection?
5. What do you think about the World Health Organization (WHO) guidelines related to ACF? (e.g. on improving early TB detection, screening guideline, screening operational guide, contact investigation)
    - Have you used any of the WHO guidelines?
    - Do you think others are using them?
    - Does anything need to be improved in these guidelines?

Engagement in ACF project, policy or scale up in specific country(ies)

6. Are you engaged in an ACF project in a particular country?
    - How are you engaged?
    - In which country are you engaged?
    - Could you describe an example(s)? (e.g. setting, risk group, activities, timeline)
7. Has this ACF project developed into/influenced policy?
    - If yes, what kind of policy? (e.g. become part of National Strategic Plan, sectoral policy or law)
    - If not, why?
8. Could you describe the/a ACF policy? (e.g. setting, risk group, activities, timeline)
9. Who was/is responsible for the different parts of the ACF policy cycle (agenda setting, policy formulation, implementation and evaluation)?
    - … in terms of human resources?
    - … in terms of collaboration?
    - … when it comes to technical input?
    - … when it comes to management?
10. Who was/is funding which part of the ACF policy cycle?
11. Which factors influence(d) the ACF policy development? / Which factors influence(d) the ACF policy implementation?
    - … thinking about the overall country context?
        i. How did it influence the ACF policy cycle?
    - … in terms of the health system and policy context?
        i. How did it influence the ACF policy cycle?
    - … when it comes to financing?
        i. How did it influence the ACF policy cycle?
    - Which factor is most powerful in influencing these processes?
12. Which other organizations/partners influence/influenced the ACF policy development? / Which other organizations/partners influence/influenced the ACF policy implementation?
    - … Donors (e.g. Global Fund, bilateral donors, researchers, etc.)
        i. How did they influence the ACF policy cycle?
    - … Funding mechanisms, specific funds, existing resources
        i. How did they influence the ACF policy cycle?
    - … International technical agencies (WHO, Stop TB Partnership, etc.)
        i. How did they influence the ACF policy cycle?
    - … Other organizations (civil society organization, non-governmental organizations, etc.)
        i. How did they influence the ACF policy cycle?
    - Which organization/partner is most powerful in influencing these processes?
13. When it comes to the use of evidence in the ACF policy development process… / When it comes to the use of evidence in the ACF policy implementation process…

- … Which type of evidence was/is being used? (e.g. global, local, scientific, tacit)?
- … What type of outcomes did/does the evidence focus on? (e.g. case detection, reduce delays, treatment outcomes, cost-effectiveness)
- … In which part of the policy cycle was/is evidence used (e.g. priority-setting, policy formulation, policy implementation, policy evaluation)?
- … What are the opportunities in using evidence for improving ACF policy cycle?
- … What are the challenges in using evidence for improving the ACF policy cycle?

14. What is your most important lesson learned related to ACF?
15. What would you like to say about future ACF policy?
16. Do you have any additional comments?
