# Supplementary material for: ‘Power plays plus push’: experts’ insights into the development and implementation of active tuberculosis case-finding policies globally, a qualitative study
Source: BMJ Open. 2020 Jun 3;10(6):e036285. doi: 10.1136/bmjopen-2019-036285 (PMC7282330; doi:10.1136/bmjopen-2019-036285)
Supplement: Author's manuscript [file bmjopen-2019-036285.draft_revisions.pdf]

BMJ Open

BMJ Open is committed to open peer review. As part of this commitment we make the peer review history of every article we publish publicly available.

When an article is published we post the peer reviewers' comments and the authors' responses online. We also post the versions of the paper that were used during peer review. These are the versions that the peer review comments apply to.

The versions of the paper that follow are the versions that were submitted during the peer review process. They are not the versions of record or the final published versions. They should not be cited or distributed as the published version of this manuscript.

BMJ Open is an open access journal and the full, final, typeset and author-corrected version of record of the manuscript is available on our site with no access controls, subscription charges or pay-per-view fees (http://bmjopen.bmj.com).

If you have any questions on BMJ Open's open peer review process please email info.bmjopen@bmj.com

BMJ Open

"Power plays plus push": Experts' insights into the development and implementation of active tuberculosis case-finding policies

Journal:	*BMJ Open*
Manuscript ID	bmjopen-2019-036285
Article Type:	Original research
Date Submitted by the Author:	09-Dec-2019

Complete List of Authors:	Biermann, Olivia; Karolinska Institutet, Public Health Sciences<br>Atkins, Salla; Tampere University, New Social Research and Faculty of Social Sciences; Karolinska Institute, Department of Public Health Sciences<br>Lönnroth, Knut; Karolinska Institutet Department of Public Health Sciences,<br>Caws, Maxine; Liverpool School of Tropical Medicine, Department of Clinical Sciences<br>Viney, Kerri ; Karolinska Institute, Public Health Sciences

Keywords:	PUBLIC HEALTH, Tuberculosis < INFECTIOUS DISEASES, QUALITATIVE RESEARCH, Health policy < HEALTH SERVICES ADMINISTRATION & MANAGEMENT

SCHOLARONE™
Manuscripts

I, the Submitting Author has the right to grant and does grant on behalf of all authors of the Work (as defined in the below author licence), an exclusive licence and/or a non-exclusive licence for contributions from authors who are: i) UK Crown employees; ii) where BMJ has agreed a CC-BY licence shall apply, and/or iii) in accordance with the terms applicable for US Federal Government officers or employees acting as part of their official duties; on a worldwide, perpetual, irrevocable, royalty-free basis to BMJ Publishing Group Ltd ("BMJ") its licensees and where the relevant Journal is co-owned by BMJ to the co-owners of the Journal, to publish the Work in this journal and any other BMJ products and to exploit all rights, as set out in our licence.

The Submitting Author accepts and understands that any supply made under these terms is made by BMJ to the Submitting Author unless you are acting as an employee on behalf of your employer or a postgraduate student of an affiliated institution which is paying any applicable article publishing charge ("APC") for Open Access articles. Where the Submitting Author wishes to make the Work available on an Open Access basis (and intends to pay the relevant APC), the terms of reuse of such Open Access shall be governed by a Creative Commons licence – details of these licences and which Creative Commons licence will apply to this Work are set out in our licence referred to above.

Other than as permitted in any relevant BMJ Author's Self Archiving Policies, I confirm this Work has not been accepted for publication elsewhere, is not being considered for publication elsewhere and does not duplicate material already published. I confirm all authors consent to publication of this Work and authorise the granting of this licence.

For peer review only

"Power plays plus push": Experts' insights into the development and implementation of active tuberculosis case-finding policies

Olivia Biermann[1]* (olivia.biermann@ki.se)

Salla Atkins[1 2] (salla.atkins@tuni.fi)

Knut Lönnroth[1] (knut.lonnroth@ki.se)

Maxine Caws[3 4] (mcaws@hotmail.com)

Kerri Viney[1 5] (kerri.viney@anu.edu.au)

1 Department of Global Public Health, Karolinska Institutet, Tomtebodavägen 18a, 17177 Stockholm, Sweden

2 New Social Research and Global Health and Development, Faculty of Social Sciences, Tampere University, Kalevantie 4, 33100 Tampere, Finland

3 Department of Clinical Sciences, Liverpool School of Tropical Medicine, Pembroke Pl, Liverpool L3 5QA, United Kingdom

4 Birat Nepal Medical Trust, Lazimpat, Kathmandu, Nepal

5 Research School of Population Health, College of Health and Medicine, Australian National University, Canberra ACT 2600, Australia

*Corresponding author

For peer review only - http://bmjopen.bmj.com/site/about/guidelines.xhtml

Abstract

Objective

To explore factors that influence national and global ACF policy development and implementation, and the use of evidence in these processes from the perspective of experts.

Design

Exploratory study based on semi-structured expert interviews.

Participants

A purposive sample of 39 experts from international, non-governmental and non-profit organizations, funders, government institutions, international networks, think tanks, universities and research institutions worldwide were conducted. Framework analysis was applied.

Results

This study accentuated the perceived need among experts for different types of evidence for ACF policy development and implementation, and for stakeholder engagement including researchers and policymakers to foster evidence use. Interviewees stressed the influence of government, donor and non-governmental stakeholders in ACF policy development. Such key stakeholders also influence ACF policy implementation, in addition to available systems and processes in a given health system, and implementers' motivation and incentives. According to the interviewees, the WHO guidelines for systematic screening face the innate challenge of providing guidance to countries across the broad area of ACF in terms of target groups, settings and screening algorithms. The guidelines could be improved by focusing on what

For peer review only - http://bmjopen.bmj.com/site/about/guidelines.xhtml

should be done rather than what *can* be done in ACF, and by providing *how to* examples. Leadership, integration into health systems and long-term financing are key for ACF to be sustainable.

Conclusions

We generated new knowledge regarding ACF policy processes globally, particularly regarding facilitators for and barriers to ACF policy development, evidence need and use, and donor organizations' influence. According to expert participants, national and global ACF policy development and implementation can be improved by broadening stakeholder engagement. Meanwhile, using diverse evidence to inform ACF policy development and implementation could mitigate the "power plays plus push" that might otherwise disrupt and mislead these policy processes.

Key words

Tuberculosis, active case-finding, community, policy development, policy implementation, evidence use

Article summary

Strengths and limitations of this study

- Expert interviews allowed a unique insight into ACF policy development and implementation from a wide range of experts' perspectives.
- New knowledge was generated, in particular about factors influencing ACF policy development and the use of evidence in ACF policy processes.

For peer review only - http://bmjopen.bmj.com/site/about/guidelines.xhtml

- Women and interviewees from low- and middle-income countries were underrepresented in the study.

Manuscript (6,813 words)

Background

Tuberculosis (TB) is a major global health emergency, especially in low- and middle-income countries. TB is curable and preventable. Still, it remains the leading cause of death from a single infectious agent and one of the top 10 causes of death worldwide [1]. In 2019, there was a gap of three million between estimated incident TB cases and those notified globally, reflecting a combination of underreporting of detected TB cases and underdiagnosis, specifically in countries with major financial and geographic barriers to accessing care [1]. Many people with TB are diagnosed only after long delays [2-4], causing increased morbidity, much suffering and economic hardship, and sustaining transmission [1].

The World Health Organization (WHO) End TB Strategy [5] was endorsed by member states at the World Health Assembly in 2014, while the United Nations Sustainable Development Goals [6] were adopted in 2015. Both aim at ending the global TB epidemic. Subsequently, there has been increasing international attention on TB. In 2017, the Global Ministerial Conference on Ending TB in the Sustainable Development Era took place in Russia, with the aim of accelerating implementation of the End TB Strategy [7]. In 2018, the UN held the first-ever General Assembly high-level meeting on TB in New York, which endorsed a political declaration to speed up progress towards ending TB. This declaration was adopted by the General Assembly on 10 October 2018 [8]. Both the Global Ministerial Conference and the General Assembly re-emphasized the importance of active case-finding (ACF).

For peer review only

Ending TB will require intensified activity to increase TB case detection [5]. One strategy for increased TB case detection is systematic screening, which is defined by the WHO as the "systematic identification of people with suspected active TB, in a predetermined target group, using tests, examinations or other procedures that can be applied rapidly" [9]. ACF is synonymous with systematic screening for active TB, although it usually implies screening outside of health facilities. ACF is mostly provider-initiated. It may target people who do not seek appropriate health care because they: a) do not have or recognize symptoms, b) do not perceive that they have a health problem requiring medical attention, or c) face barriers in accessing appropriate care [9].

ACF has been implemented for decades primarily in high-income countries, starting with mass screening campaigns in the general population in the 1950s and 1960s, then moving towards specific risk populations in recent decades, such as migrants from high-incidence countries and prison populations [10,11]. In low- and middle-income countries, the interest in ACF has increased in recent years, mainly as a response to a sustained case detection gap documented in TB prevalence surveys, annual Global TB Reports produced by WHO [1] and the development of new WHO guidelines on systematic screening [9].

Questions remain about both *if* ACF in general is worthwhile, as well as *how* to best develop and implement ACF in a given context as a synergistic, rather than parallel structure to the given health system. The evidence base is weak concerning the benefits and cost-effectiveness of ACF on both individual and community levels and how these vary between target risk groups [12]. However, potential benefits of ACF for patients include reduced morbidity, mortality and socioeconomic consequences due to earlier diagnosis, while society can benefit from reduced transmission and a reduced burden of TB, which often affects the most economically productive members of a society [9]. There is some evidence that TB screening in high-risk groups can significantly increase TB case notifications [13-15]. However, from

For peer review only - http://bmjopen.bmj.com/site/about/guidelines.xhtml

the health system perspective screening can be costly and lead to diversion of scarce resources. It can also cause harm to patients, e.g. by increasing the risk of false positive diagnoses, creating an additional financial burden associated with attending screening and follow-up, or increased stigma and discrimination, if not properly targeted and implemented [16].

The potential benefits and challenges of ACF need to be carefully balanced when designing and implementing ACF. Given the relatively weak evidence base for ACF, related policy development and implementation processes rely on stakeholders' tacit knowledge, values and preferences. Yet, little is known about the latter, which potentially impact the development and implementation of national and global ACF policies. The aim of this study was to explore the views of experts on the factors that influence ACF policy development and implementation, and their views of the use of evidence in these processes.

Methods

This was an exploratory study based on semi-structured expert interviews [17]. The research team used the COREQ (COnsolidated criteria for REporting Qualitative research) Checklist [18] to report the study (*Additional file 1*).

Recruitment and sample selection

The interviewees were purposively sampled to include stakeholders involved in ACF policy development and implementation based at international (n=16), non-governmental (n=2) and non-profit organizations (n=2), funders (n=4), government institutions (n=2), international networks (n=2), think tanks (n=1), universities (n=6) and research institutions (n=3), as well as one independent consultant. The research team compiled the initial list of interviewees

For peer review only - http://bmjopen.bmj.com/site/about/guidelines.xhtml

based on knowledge of networks of experts and on the published scientific literature. The list was discussed with, expanded and verified by two independent experts in the field.

OB contacted 50 individuals via email. Of these, two suggested that their colleagues be interviewed instead, eight did not reply and one declined participation due to lack of time and interest. Table 1 provides an overview of the 39 participants who agreed to participate, their sex, professional affiliation and country where they are currently based, classified according to the World Bank [19]. In the results section, we have used quotes from interviewees across all country income levels to increase the dependability of the results [20]. Moreover, where possible in the results, we have tried to reflect all participants' voices.

Table 1. Participants and their background information (in chronological order)

ID	Sex	Affiliation	Country classification according to the World Bank [19]
1	M	University	High-income country
2	M	International organization	Low-income country
3	M	Government institution	Low-income country
4	M	International organization	Low-income country
5	M	Government institution	Low-income country
6	M	International organization	Low-income country
7	M	Non-governmental organization	Low-income country
8	M	Non-governmental organization	Low-income country
9	F	Research institution	High-income country
10	M	International organization	High-income country

For peer review only - http://bmjopen.bmj.com/site/about/guidelines.xhtml

11	M	International organization	High-income country
12	M	Research institution	High-income country
13	F	Non-profit organization	Upper middle-income country
14	F	International network	Lower middle-income country
15	M	Funder	High-income country
16	M	International organization	High-income country
17	F	International organization	High-income country
18	M	Research institution	High-income country
19	M	International organization	High-income country
20	M	University	High-income country
21	M	University	High-income country
22	M	International network	High-income country
23	F	Think tank	High-income country
24	F	International organization	High-income country
25	M	International organization	High-income country
26	M	International organization	High-income country
27	M	Independent consultant	Lower middle-income country
28	M	International organization	High-income country
29	M	International organization	Lower middle-income country
30	M	Funder	High-income country
31	M	Funder	Lower middle-income country
32	M	University	High-income country
33	M	Funder	High-income country
34	M	International organization	Lower middle-income country

For peer review only - http://bmjopen.bmj.com/site/about/guidelines.xhtml

35	M	International organization	High-income country
36	F	University	Low-income country
37	M	University	High-income country
38	M	Non-profit organization	High-income country
39	M	International organization	Upper middle-income country

Data collection

OB collected the data between February and May 2018 through semi-structured interviews in conversational form via the phone or in person. She developed the interview guides (*Additional file 2*) which MC, KL and KV provided feedback on. The first interview was conducted as a pilot interview after which the guide was revised by making it shorter to focus on the principal topics of interest.

OB asked the interviewees about their experience in developing and/or implementing ACF policies, factors that influenced these policy processes, and the use of evidence. The interviews were audio-recorded. No repeat interviews were carried out and no formal field notes were taken. In order to capture opinions from the diverse range of experts involved in ACF policy development and implementation, OB conducted interviews beyond reaching information power [21]

Eleven interviews were carried out face-to-face; out of these, eight interviews were conducted during a field visit to Nepal, two during WHO meetings and one at an international organization. During the interviews, only OB and the respective interviewee were present. The typical duration of an interview was 30-60 minutes. The audio-recorded interviews were transcribed verbatim. The anonymity and confidentiality of the participants was ensured by

For peer review only - http://bmjopen.bmj.com/site/about/guidelines.xhtml

181 coding the participants as numbers and removing all identifiers except the respondent
182 affiliation in the presentation of the results. OB offered all participants the opportunity to view
183 their transcripts for comments or correction, however, only a handful of participants requested
184 to see the transcripts. No comments or corrections were made by those who chose to view the
185 transcripts.

186

187 OB is a doctoral student in public health sciences focusing on ACF and with experience in
188 qualitative research. The multidisciplinary research team consisting of a medical doctor, an
189 epidemiologist, a microbiologist and a social scientist helped elicit the diverse perspectives on
190 ACF policy development and implementation.

191

192 *Data analysis*

193 OB analysed the qualitative data from the expert interviews with *NVivo 11* using framework
194 analysis [17]. The data was analysed abductively; identifying themes a priori, while allowing
195 for additional themes to emerge from the data. Using the framework analysis approach as
196 described by Gale et al. [22], OB coded all interviews and developed an analytical framework.
197 SA and KV provided comments on the coding, based on which OB revised the codes. The
198 data was then charted into a framework matrix, on which SA and KV provided feedback. OB
199 interpreted the data by writing memos for each study theme, and discussed these with SA, KL
200 and KV. Table 2 provides an example of the coding process.

201

202 *Table 2. Example of the coding process*

**Interviewee**	**Quote**	**Code**	**Category**	**Theme**
I-27, independent consultant in a	"*So, I think it's the political push that then*	Government influencing	Government leadership and	Factors influencing

For peer review only - http://bmjopen.bmj.com/site/about/guidelines.xhtml

lower middle-income country	*forces the technocrats to develop policies."*		commitment	ACF policy development

Patient and public involvement

The preliminary findings were shared at different scientific conferences in 2018, such as the Global Symposium on Health Systems Research, the First Annual Conference on Implementation Science and Scale-up and the World Union Conference on Lung Health. The interaction with participants of these events provided unique opportunities for validating the findings. For the presentation of preliminary findings at the Union conference, personalized invitations were sent to all 39 interviewees. A few interviewees attended and provided feedback. As such, the presentation of preliminary findings gave an opportunity for member-checking. No direct changes were made based on the validation and member-checking, but these processes helped to more critically reflect on the findings. Once published, the results of this study will be reported back to each interviewee individually. In addition, targeted issue briefs will be developed for researchers and decision-makers in the field. We will also share the results with the public via a video and short messages on social media.

Results

We generated the following themes from the data: 1) evidence generation and use, 2) factors influencing ACF policy development, 3) factors influencing ACF policy implementation, 4) WHO guidelines on systematic screening and 5) sustainability of ACF. Table 3 provides an overview of the 5 main themes and the 16 related codes. The benefits and risks of ACF were additional major themes which will be analyzed and discussed in a separate publication. Overall, the interviewees had a wide variety of views on ACF; from ACF being a "waste

For peer review only - http://bmjopen.bmj.com/site/about/guidelines.xhtml

basket” for resources to it being “common sense”.

Table 3 Summary of major themes and categories related to ACF policy development and implementation

1	Evidence generation and use	1	Dissemination and exchange of evidence
		2	Demand for evidence by decision-makers
		3	Stakeholder engagement to facilitate evidence use
2	Factors influencing ACF policy development	1	Government leadership and commitment
		2	Donor funding
		3	Non-governmental organizations’ experience
3	Factors influencing ACF policy implementation	1	Human and financial resources
		2	Systems, processes and resources to build on
		3	Donor funding and related target-setting
		4	Government power
		5	Health workers’ motivation and incentives
4	WHO guidelines on systematic screening	1	Positive and negative perceptions
		2	Contextualization of global guidelines locally
		3	Suggested improvements
5	Sustainability of ACF	1	Opportunities for sustainability
		2	Challenges for sustainability

Theme one: Evidence generation and use

Most interviewees described the evidence on ACF as being relatively limited and emphasized the need to generate different types of evidence to inform ACF policy development and

For peer review only

implementation. They stressed the importance of disseminating and exchanging evidence, of the demand for evidence by decision-makers and stakeholder engagement to enable evidence use. Apart from highlighting specific types of evidence, interviewees across the different settings had similar views with regards to this theme.

Interviewees underlined that a variety of evidence is needed and demanded by decision-makers working on ACF; from effectiveness and health economic evaluations to implementation and operational research. One interviewee from a university in a high-income country stressed that to demonstrate effectiveness, there is a need *"to do ACF in the context of randomized controlled trials"* (I-32). Another interviewee from a non-governmental organization (NGO) in a low-income country highlighted the importance of distinguishing clearly where the decisions are being made; be it at the community, district or national level: *"I think this is very important, i.e. what types of evidence you would need to make decisions at various levels (…). What evidence is enough evidence at what level to take the decision"* (I-7). Local evidence was said by many to play a significant role, e.g. about available health and diagnostic facilities, and health workers' capacity and experience in communicating with communities. In particular, evidence from national TB prevalence surveys was described as significant for TB policy development more broadly. Two interviewees from funding organizations in high-income countries concluded that countries should be encouraged *"to adopt [ACF] policies based on the local evidence and then move forward, rather than waiting for systematic reviews"* (I-30) and *"you should implement enough to figure out what's practicable and what works, and then that should become policy"* (I-15).

According to the interviewees, evidence use in ACF policy development and implementation necessitates evidence dissemination and exchange, especially to share unpublished findings.

For peer review only - http://bmjopen.bmj.com/site/about/guidelines.xhtml

One interviewee from an international organization highlighted: *"Unfortunately, we are [from a low-income country] and we are not very good at publishing. We've got a wealth of experience that is unpublished (...) but it has been presented at several conferences."* (I-35). Depending on the country context, gaps may exist between evidence and policy and/or between policy and practice. As one interviewee from an international organization in a high-income country pointed out: "*Countries are different. As I said, in [that country] (...) from evidence to policy was difficult. But once it [ACF] was inside the policy or even without the policy, they used to easily convert it to practice. But here [in our country] (...) evidence to policy is easier, but policy to practice is more difficult."* (I-28)

Interviewees emphasized that researchers should engage with key stakeholders from the beginning of the research process to foster research use in ACF policy development and implementation; stakeholders may include the WHO, the Ministry of Health and the National TB Programme (NTP). *"Make sure that you have the right partners from the beginning; partners who are going to take your results and actually do something with them. Because otherwise you are kind of doing it [research on ACF] in a vacuum",* said one interviewee from a university in a high-income country (I-37). Moreover, to spark dialogue through stakeholder engagement, an interviewee from an NGO in a low-income country stressed that one must *"create platforms, or you need to use the platforms which are already there."* (I-7) Regular review meetings at sub-national and national levels to discuss challenges and successes related to ACF offer one such platform. Overall, evidence use was said to be influenced by *who* is being engaged and by personal contacts which may be *"more important than they should be",* as another interviewee from a university in a low-income country described (I-36).

For peer review only

Theme two: Factors influencing ACF policy development

According to the interviewees, many different stakeholders influence ACF policy development, specifically governments, donors and NGOs. Interviewees underlined stakeholder involvement as being necessary for policy development and the contextualization of global policy into local realities. Interviewees did not have any contradicting views with regards to this theme, but rather highlighted the specific roles of certain stakeholders they thought were most influential in ACF policy development.

The leadership, buy-in and commitment of governments and NTPs, were described as being vital for ACF policy development and implementation. India was mentioned as a prime example where political push *"forced the technocrats to develop policies and implement them"* (I-27, an independent consultant in a lower middle-income country). Governments make decisions for political reasons or donor incentives, even if these contradict the evidence. One representative from an international organization in a high-income country highlighted an example of action perceived to be contradicting their view of the evidence: "*Women and children of reproductive age (...) should only be included as part of the passive system not as a priority for ACF ever. But when you talk to NTP managers, there is strong political pressure and a perception that donors want them to focus on women and children." (I-24)*

Donor organizations such as the Global Fund and the case-finding initiative TB REACH (the latter is coordinated by the Stop TB Partnership and funded largely by Global Affairs Canada) were described as being influential in ACF policy development, e.g. TB REACH was said to have *"brought this concept of ACF to the country"* (I-2, interviewee from an international organization in a low-income country), while the Global Fund *"hold[s] every power to*

For peer review only - http://bmjopen.bmj.com/site/about/guidelines.xhtml

change things and not to change things" regarding ACF policy development (I-7, representative from an NGO in a low-income country). Likewise, interviewees pointed out that donors' influence was linked to WHO's influence, as donors request countries to adopt WHO guidelines to be eligible for funding: "*Why national policymakers are looking mainly at things like WHO documents: because a lot of them get Global Fund money and Global Fund money is often aligned with countries implementing WHO policies",* described an interviewee who is based at a research institution in a high-income country (I-9). This observation was shared by another interviewee, from an NGO in low-income country (I-8). Linking back to the preceding theme on evidence generation and use, it seemed from the interviews that it was important to acknowledge that TB REACH projects have the potential to generate useful evidence for future policy and practice, as an independent consultant in a lower middle-income country pointed out (I-27).

Interviewees said that NGOs are often the "implementers" of ACF whose years of experience are of great value for ACF policy development and they should therefore be involved in the same, e.g. in policy dialogues with the government and other key stakeholders. One representative of an NGO in a low-income country stated: "*We [NGOs] are the one who really deal with the people, really deal with the community. We have the evidence. We have the good photographs. We have the data. We have a number. And, if I can speak very nicely in the presentation, data has to speak and that data is brought by the hard-working, my dear friends working in the field level. We are the ones who can influence [ACF policy development]."* (I-8)

Theme 3: Factors influencing ACF policy implementation

Interviewees elaborated on available resources, systems, processes and resources within a

For peer review only - http://bmjopen.bmj.com/site/about/guidelines.xhtml

given health system, donor and government stakeholders, as well as the motivation and incentives for health workers as major factors influencing ACF policy implementation. Interviewees emphasized the role of particular stakeholders, as well as barriers and facilitators they thought were most influential in ACF policy implementation, while no clearly contradictory views on this theme emerged.

The implementation and scale-up of ACF policies depends on the availability of financial resources, as many interviewees stressed. *"We realized that in a country like [our country], we have great policies. The problem is the implementation. So, getting it done is always problematic. And this is where the support of the development partners, funded through PEPFAR, have been key to implement these policies, particularly ACF policies",* one interviewee described (I-39, interviewee from an international organization in an upper middle-income country). ACF implementation may *"just stop because [there is] no funding"* (I-29, interviewee from an international organization in a lower middle-income country). An interviewee from a funding organization in a high-income country provided a different perspective regarding the funding for ACF by highlighting that *"ACF through government funding can be more difficult than doing it through donor funding"* (I-15). This perspective may inhibit long-term thinking about ACF, as it seems to focus on immediate action to implement rather than sustainability, which is more likely to come with government investment. In addition to limited financial resources, human resource constraints for ACF were highlighted as a major challenge by experts from low-, middle- and high-income countries. These constraints could hinder NTPs in thinking more strategically and ambitiously about how to address TB comprehensively.

The use of existing systems and processes in a given health system was said to be central

For peer review only - http://bmjopen.bmj.com/site/about/guidelines.xhtml

because *"if you start from scratch, it [ACF] is much more difficult than if there are already things to which you can link"*, as an interviewee from an international organization in a high-income country pointed out (I-17). Interviewees mentioned that ACF policy implementation can build upon experience from existing screening programmes (e.g. cervical cancer screening), activities for vulnerable populations (e.g. needle exchange programmes), health care infrastructure (e.g. chest X-ray busses), already known locations for screening in high incidence areas and trained human resources (e.g. those involved in prevalence surveys). Yet, pursuing synergies may be challenging due to the fragmentation of activities. In addition, the structure and financing of TB within a health system matters in terms of availability of resources: *"TB has tended to fall into the preventative [arm of the health system] and that has limited the availability for resources"*, described an interviewee from a university in a high-income country (I-32).

Processes including supportive supervision, monitoring and the use of Standard Operating Procedures are critical for ACF policy implementation and are necessary to avoid corruption, interviewees discussed. In one country, the *"whole case-finding system collapsed along with the supervision"* (I-16, interviewee from an international organization in a high-income country). Moreover, processes that strengthen communication with, engagement of and awareness-raising among communities were described as instrumental for ACF policy implementation, e.g. to help reduce stigma. One interviewee from a university in a high-income country mentioned how the community *"has started to advocate loudly for ACF services"* (I-20).

Many interviewees underlined that donors influence the implementation of ACF policies in countries with no or insufficient domestic resources. *"The piper will determine what music*

For peer review only - http://bmjopen.bmj.com/site/about/guidelines.xhtml

you play" (I-35, interviewee from an international organization in a high-income country), which, again, highlights the power which donor organizations are perceived to have in influencing ACF policy implementation, and the possible resulting lack of a sense of policy ownership in some countries. Donors influence ACF policy implementation by setting targets for their funding recipients and pushing them towards reaching them. One interviewee from an international organization in a high-income country described: *"These targets that countries have set, that donors have set; people are very anxious (…) and that often means the easiest short cut is to do ACF, even if it is a little bit unethical or little bit using low specificity tools, so you have a little bit of over diagnosis. Donors are very comfortable with that."* (I-24) The consequences of implementing ACF under donor pressure are unclear, and should be balanced against the unethical nature of inaction on the TB epidemic, but scale-up of inaccurate diagnostic strategies might lead to heightening the potential risks of ACF such as increasing false-positive diagnoses, as the interviewee mentioned.

ACF policy development and implementation depend on *"power plays plus push"*, e.g. in a country with no written ACF policy, ACF was still being implemented because the NTP manager was respected and able to push for it (I-29, interviewee from an international organization in a lower middle-income country). The aforementioned pressure by politicians, donors and WHO may be seen as additional examples of "power plays plus push". Many interviewees highlighted the important role of power dynamics in ACF policy implementation. It seems crucial to be aware of such dynamics, while the use of evidence may help mitigate them. ACF policy implementation is in itself a balancing act, which power imbalances might negatively impact.

The motivation of health workers and volunteers is an important enabler for ACF policy

For peer review only - http://bmjopen.bmj.com/site/about/guidelines.xhtml

implementation. These "implementers" can be strongly motivated by their desire to help people, by understanding the benefit of ACF for communities, by receiving feedback on the outcomes of their work (e.g. using performance indicators) and/or by feeling ownership of the ACF process, according to the interviewees. Financial and non-financial incentives (e.g. salaries, transportation allowances, provision of motorbikes or mobile airtime) have a significant role in motivating health workers and volunteers to implement ACF as an outreach activity, interviewees discussed. Nevertheless, incentives can raise expectations and distort ACF policy implementation in the long-term, e.g. if government health workers are paid extra as part of an ACF project, they will also expect an extra pay for such activities in the future and for other work; another balancing act. While incentives should be in line with what a country could adopt later, they are often difficult or impossible for governments to sustain, an interviewee said.

Theme 4: WHO guidelines on systematic screening

This theme focuses on stakeholders' perceptions of the WHO guidelines on systematic screening, the need for their contextualization and suggestions for improving them. This theme elicited different views among stakeholders, which are described in the following.

The WHO guidelines on systematic screening are perceived positively by many, e.g. as a reference document when planning ACF activities as well as to put ACF on the agenda. Positive perceptions of the guidelines were described by interviewees from different countries, while negative perceptions were only voiced by interviewees in high-income countries. Such negative perceptions included the guidelines being vague, lacking information about the *how-to* of ACF and being unduly negative in terms of mentioning the risk of increasing false-positive diagnoses through ACF. Low-income countries may be more

For peer review only - http://bmjopen.bmj.com/site/about/guidelines.xhtml

receptive to and reliant on WHO guidelines, while in a middle-income country *"you've got really serious domestic universities providing the formal policy evidence. And the country kind of says 'Thanks but no thanks' to outside opinions. They are really driving their own decisions. WHO is really not consulted very much, if at all",* a representative of a funding organization in a high-income country described (I-15). An interviewee from an international organization in a high-income country said: *"When you have something that is so broad – and you're talking about ACF which can be so many different things – it's just very hard to have something that works the same way in different countries (…). I think that's the main shortcoming around the guidance."* (I-28)

Interviewees emphasized the necessity of contextualizing the WHO guidelines on systematic screening, e.g. depending on a country's income level, epidemiology and availability of diagnostic tools. One interviewee from a funding organization in a high-income country pointed out that *"you just can't be as prescriptive and exact as you are in the more clinical guidelines*" (I-15), which seems like an important observation and reminder about the limitations that ACF policies will always have. According to the interviewees, contextualization of guidelines can happen in a stepwise approach, e.g. a country pilots the use of a guideline before adopting and adapting it.

Review meetings with WHO and other partners can provide a platform for discussions around guideline adaptation, interviewees said. Yet, countries have faced challenges in contextualization, e.g. WHO recommends using chest X-ray which was too expensive in a country and could thus not be used (I-4, from an international organization in a low-income country). In another instance, WHO describes how contacts of an index TB patient should provide their address, while individuals were hesitant to do so due to the stigma surrounding

For peer review only - http://bmjopen.bmj.com/site/about/guidelines.xhtml

458 TB in the country (I-14, interviewee from an international network in a lower middle-income
459 country). More support for the contextualization of guidelines may be needed.

460

461 The interviewees suggested that the WHO guidelines for systematic screening [9] must be
462 updated based on new evidence, e.g. evidence from prevalence surveys, gender analyses,
463 studies about specific risk groups (e.g. drug users and indigenous populations) and what
464 works and how this works, with regards to ACF. In this process, WHO should be aware of
465 and avoid conflicts of interest. This comment is in line with what an interviewee previously
466 highlighted about the role of personal contacts to bridge the research-policy gap. These types
467 of biases may undermine the integrity of the process and the resulting quality of guidelines
468 and policies.

469

470 Some interviewees lamented that WHO can be paralyzed by the need to use the strongest
471 evidence available and suggested that the organization should consider more programmatic,
472 less scientifically rigorous data. One interviewee from a university in a high-income country
473 described: *"Usually we're relying very heavily on WHO for global policy using the GRADE*[1]
474 *approach with the PICO*[2] *and all that stuff. I think that's laudable, but sometimes I find that*
475 *weird, subjected to the tyranny of the great process, and you don't make progress in smaller*
476 *areas with a paucity of evidence."* (I-21)

477

478 In addition, interviewees pointed out that WHO recommendations should be based on what

[1] GRADE (Grading of Recommendations Assessment, Development, and Evaluation) was developed for creating summaries of research evidence to help guide health decision-making. It is currently the most widely used tool for evaluating the quality of science, with more than 110 organizations endorsing the method [23].
[2] The PICO acronym stands for: P – Patient, Problem or Population; I – Intervention; C – Comparison, control or comparator; O – Outcome(s) (e.g. pain, fatigue, nausea, infections, death). The PICO process (or framework) is a mnemonic used in evidence-based practice to frame and answer a clinical or health care related question. The PICO framework is also used to develop literature search strategies, e.g. in systematic reviews [24].

For peer review only - http://bmjopen.bmj.com/site/about/guidelines.xhtml

should be done, not on what *can* be done. For example, countries (not WHO) have to be the ones to decide about their ability to pay for Xpert MTB/RIF® as a diagnostic tool. This point of view illustrates a stark contrast to the contextualization challenges mentioned above, e.g. where the use of X-ray was recommended, but, frustratingly, was unable to be applied in a country as it was not feasible to implement. Moreover, the WHO guidelines could be improved by not only describing the *what,* but the *how* of systematic screening including ACF, many interviewees said. One interviewee from an NGO in a low-income country suggested: *"You can come up with different scenarios: 'If the context is this, then...', 'If the context is that, then…'. (…) Unless guidelines presents [the] 'how' better – how and who would do that, how long would it take – then it's meaningless."* (I-7)

Theme 5: Sustainability of ACF

The sustainability of ACF was a cross-cutting theme in this analysis. Interviewees elaborated on opportunities and challenges related to sustainability. *"TB is not a like smallpox or polio. It's a long-term sustainable (…) matter",* an independent consultant in a lower middle-income country described (I-27). That is, even more perseverance and long-term thinking may be required to end TB. Interviewees expressed similar views and concerns regarding this theme.

Interviewees highlighted that the interest in and leadership for ACF through the government and the NTP are important for the sustainability of ACF. Additionally, the sustainability of ACF requires its integration in and funding through the given health system. An interviewee from an international organization in a low-income country described: *"If this [ACF] were to be sustainable, it should start with the initiation of the NTP. (…) It has to be supported, facilitated, monitored. Because it is actually the NTP which later needs to uptake that, if it is effective and it's also NTP's role to monitor what's happening." (I-2)* Many interviewees

For peer review only - http://bmjopen.bmj.com/site/about/guidelines.xhtml

highlighted the important role of NTPs. We have also conducted a cross-sectional survey with NTP managers from the 30 high-burden TB countries which will shed light on their views on ACF policy development and implementation, including the sustainability of ACF (manuscript in preparation).

The sustainability of ACF may be restricted in places with frequent government and staff turnover, which makes it difficult to get long-term commitment for ACF from decision-makers, interviewees stressed. Of course, such turnover will affect areas beyond ACF. Also, ACF cannot be sustainable, if it depends on donor funding. One interviewee from an international organization in a high-income country summarized the situation as follows: *"It [ACF] is difficult to sustain. Most of the activities that have been done for ACF have been project-based. (…) So, the Global Fund comes and says: 'Here is a pot of money for ACF for the next three years.' (…) And then the USAID comes and says: 'We will run this project that is forecasting on ACF. We should do it for five years.' And then the money goes. Or TB REACH comes and says: 'Here you have 1 million dollars to find cases of TB, chase them all over the villages and tell us how many you get with this money.' And people do it. But that's not a sustainable way of doing this and this should be part and parcel of routine programming."* (I-35)

Discussion

In summary, this study accentuated experts' perceived need for different types of evidence for ACF policy development and implementation, and for stakeholder engagement to foster evidence use. Interviewees stressed the influence of government, donor and NGO stakeholders as influential players in ACF policy development. Such key stakeholders also

For peer review only - http://bmjopen.bmj.com/site/about/guidelines.xhtml

influence ACF policy implementation, in addition to available systems and processes in a given health system and implementers' motivation and incentives. The WHO guidelines for systematic screening were said to face the innate challenge of covering the broad area of ACF in terms of target groups, settings and screening algorithms. Interviewees suggested that the guidelines could be improved by incorporating new and different types of evidence, by focusing on what *should* be done rather than what *can* be done, and by providing examples of the *how* of ACF. Finally, for ACF to be sustainable, interviewees stressed the need for leadership for ACF, its integration into health systems and the transition from donor to government funding.

Strengths and limitations

While the available evidence in this area often focuses on ACF policy implementation [25], this study fills important knowledge gaps by identifying factors influencing ACF policy development and characterizing evidence use in ACF policy development and implementation, from the perspective of experts in the field. Moreover, this study offers an increased understanding of donor organizations' influence on ACF policy processes. The number and diverse range of experts involved in this study, as well as the member-checking carried out, increase the study's trustworthiness, including its confirmability and transferability [20]. The transferability of this study's results may be limited given that only a minority of the experts were from low- and middle-income countries (38%; 15 out of 39 experts). Nevertheless, all had working experience from low-and middle-income countries. The results may furthermore be limited as an even smaller minority were women (18%; 7 out of 39 experts). The gender bias reflects the lack of gender parity in leadership positions in the field of global health [26]. We did not systematically conduct analyses by stakeholder group. We have highlighted the affiliations of interviewees quoted, when relevant. Due to the

For peer review only - http://bmjopen.bmj.com/site/about/guidelines.xhtml

richness of the data, we will publish a separate in-depth analysis of the perceived benefits and risks of ACF based on the expert interviews. We will furthermore complement the results of this study with a cross-sectional survey with NTP managers from the 30 high-burden TB countries, which gives in-depth insights into contextual factors and evidence use in national and local ACF policy development and implementation.

Building on a broad evidence base

Interviewees emphasized the need for a variety of evidence, such as impact and economic evaluations, operational and qualitative research. Qualitative evidence has proven essential in developing and implementing health policies including in low- and middle-income countries, e.g. to prevent and treat malaria during pregnancy [27]. In the case of ACF, decision-makers may need qualitative evidence on, for example, factors influencing participation in ACF or the retention of health workers. Likewise, qualitative evidence syntheses have emerged as an important approach to inform national and global health policy development and implementation [28] and could also be useful for improving future ACF policies.

Making and implementing better ACF policies through stakeholder engagement

Successful ACF policy development and implementation necessitate stakeholder engagement, interviewees highlighted. Stakeholder engagement is an inclusive process essential for achieving legitimate decisions, which are accepted by the population and conducive to effective implementation [29]. Specifically, interviewees stressed the importance of community engagement to enhance the implementation of ACF. Available evidence also shows the importance of community engagement and support for ACF implementation, e.g. through collaboration with respected community leaders (i.e. chiefs, civic leaders, village elders and counsellors) [30,31]. In addition, familiarity with the community [32] and

For peer review only - http://bmjopen.bmj.com/site/about/guidelines.xhtml

community buy-in [33] as well as community appreciation and respect through the engagement of community health workers were said to be important [34,35]. Stakeholder engagement is also relevant for the development of WHO guidelines at the global level, and their adaptation to the national or subnational levels, where a wide array of stakeholders with diverse sets of values should be involved [36,37].

Moving from "paralyzing" to "empowering" WHO guidance

The interviewees had many suggestions for improving the WHO guidelines on systematic screening [9], questioning the appropriateness of only using the GRADE approach in the context of ACF. The WHO guidelines make graded recommendations about screening specific risk groups for TB, including three strong recommendations[3] and four conditional recommendations[4] [9]. The conditionality makes decision-making in ACF complex by leaving recommendations open to interpretation. For example, the conditionality may "paralyze" decision-makers to move screening outside of health facilities, as ACF in many vulnerable groups is only conditionally recommended. However, despite conditional recommendations and "low-quality" or "very low-quality evidence" that all of the WHO's recommendations on systematic screening are based on [9], decision-makers must still act, either in deciding to implement or taking the decision not to. The Global Fund and TB REACH can provide guidance in interpreting the guidelines. Yet, countries should guarantee that these interpretations and adaptations are based on the local epidemiology, health system capacity, resources, feasibility, effects and economic impact, etc. This information is most likely from qualitative studies, monitoring and evaluation research and quasi-experimental studies. This

[3] Strong recommendations: Screening in household contacts and other close contacts, people living with HIV and current and former workers in workplaces with silica exposure.
[4] Conditional recommendations: Screening among prisoners, in people with an untreated fibrotic lesion seen on chest X-ray, in settings where the TB prevalence in the general population is 100/100 000 population or higher, in geographically defined subpopulations with extremely high levels of undetected TB and other subpopulations that have very poor access to health care.

For peer review only - http://bmjopen.bmj.com/site/about/guidelines.xhtml

would be paramount in order not to move away from the guidelines' original intention. Ensuring continuous monitoring and evaluation is therefore important [38]. GRADE-CERQual (Grading of Recommendations Assessment, Development and Evaluation-Confidence in the evidence from reviews of qualitative research) [39,40] may be a useful resource for future global systematic TB screening guideline development. It has been developed to assess confidence in findings from qualitative evidence syntheses. Additionally, the GRADE Evidence to Decision Framework for Health System and Public Health Decisions [38] or the WHO-INTEGRATE Evidence to Decision Framework [41] could be valuable to assess evidence for a complex intervention such as ACF.

Integrating ACF into health systems for sustainability

Interviewees underlined the need to integrate ACF into a given health system for it to be sustainable. Such integration may start with an assessment of the given health system context to understand available structures (e.g. infrastructure, budget structure and trained human resources) and processes (e.g. supportive supervision and monitoring). Interviewees described these resources as being paramount to link to and build upon. The fact that participants highlighted the need for health system integration, which seems to be relevant for any health intervention, may indicate that such integration cannot be taken for granted and/or might not always occur in ACF. It is important to acknowledge that "integration" may describe a variety of organizational arrangements across different settings [42]. Additionally, in many low-income countries, interventions generally operate through a complex patchwork of arrangements, rather than through totally stand-alone or totally integrated approaches [43].

To embed ACF into health systems, available systems for outreach and health promotion [4], laboratory networks [44] and free services [45] have been highlighted. Moreover, given the importance of community health workers for implementing ACF, their integration into the

For peer review only - http://bmjopen.bmj.com/site/about/guidelines.xhtml

health system has been emphasized [46,47]. Importantly, the collaboration between various actors has been described as key for sustainable ACF implementation. The latter includes collaboration between public health practitioners and clinicians [48], district TB teams and government health staff [49], health care staff and community health workers [32,46]. Moreover, collaboration between HIV and TB sectors [50], with laboratory staff [46] and with community organizations [50,51] has been described as important. Government, NTPs, WHO and donors, whose key roles in ACF policy development and implementation have been described by the interviewees, should contribute to the long-term thinking and long-term action related to ACF and towards ending TB. Murphy and colleagues [52] emphasize that only a mix of appropriate evidence, key stakeholders, processes and structures would be a solution for evidence-informed policy development and implementation.

Answering the "how-to" questions of ACF through future research

This qualitative study demonstrated that we know much about facilitators and barriers for ACF policy development and implementation, as well as about the need and use of evidence in these processes. Still, we know less about *how* to strengthen those facilitators, *how* to overcome those barriers and *how* to strengthen research use. Implementation research that sheds light on what works for whom and under which conditions may be particularly helpful to answer some of those *how* questions. Moreover, operational research that uses available local data, e.g. on TB notifications, may help inform local decision-making around ACF. Finally, mixed-methods studies can help explore the complexity of ACF policy development and implementation in the future, as they have the potential to both increase contextual understanding and reduce biases.

For peer review only - http://bmjopen.bmj.com/site/about/guidelines.xhtml

Conclusion

Based on a variety of experts' perspectives, we generated new knowledge regarding ACF policy processes globally, in particular regarding facilitators for and barriers to ACF policy development, evidence need and use, and donor organizations' influence. Bringing together these different views creates a more comprehensive picture of ACF policy development and implementation today and indicates ways to strengthen such processes in the future: National and global ACF policy development and implementation can be improved by broadening stakeholder engagement and ownership; from decision-makers at the Ministry of Health to community leaders and members. Meanwhile, using diverse evidence to inform ACF policy development and implementation could mitigate the "power plays plus push" that might otherwise disrupt and mislead these policy processes. Our findings complement the existing evidence base and can inform future national and global ACF policy processes.

For peer review only

References

1. WHO. Global TB Report 2019. Geneva: World Health Organization; 2019.
2. Storla DG, Yimer S, Bjune GA. A systematic review of delay in the diagnosis and treatment of tuberculosis. BMC Public Health. 2008;8(15).
3. Corbett EL, Bandason T, Duong T, Dauya E, Makamure B, Churchyard GJ, Williams BG, et al. Comparison of two active case-finding strategies for community-based diagnosis of symptomatic smear-positive tuberculosis and control of infectious tuberculosis in Harare, Zimbabwe (DETECTB): a cluster-randomised trial. Lancet 2010;376:1244-53.
4. Lönnroth K, Corbett E, Golub J, Godfrey-Faussett P, Uplekar M, Weil D, et al. Systematic screening for active tuberculosis: rationale, definitions and key considerations. Int J Tuberc Lung Dis. 2013;17(3):289-298.
5. WHO. The End TB Strategy. Global strategy and targets for tuberculosis prevention, care and control after 2015. Geneva: World Health Organization; 2015a.
6. UN. Transforming our world: The 2030 agenda for sustainable development. USA, New York: United Nations; 2015.
7. WHO. Ministerial Conference on TB, 2017. Available from: https://www.who.int/mediacentre/events/2017/ministerial-conference-tb/en/. Accessed on 22 October 2019.
8. UN. Political declaration of the high-level meeting of the General Assembly on the fight against tuberculosis. USA, New York: United Nations; 2018.
9. WHO. Systematic screening for active tuberculosis: principles and recommendations. Geneva: World Health Organization; 2013.

For peer review only - http://bmjopen.bmj.com/site/about/guidelines.xhtml

684 10. Eang MT, Satha P, Yadav RP, Morishita F, Nishikiori N, van-Maaren P, et al. Early detection of tuberculosis through community-based active case finding in Cambodia. BMC Public Health. 2012;12:469. doi: 10.1186/1471-2458-12-469.

687 11. Tyler PJ. 'The Celluloid Strip' – Mass screening for Tuberculosis in New South Wales, 1950-1975. Journal & Proceedings of the Royal Society of New South Wales. 2003;136:29-43.

690 12. Kranzer K, Afnan-Holmes H, Tomlin K, Golub JE, Shapiro AE, Schaap A, et al. The benefits to communities and individuals of screening for active tuberculosis disease: a systematic review. Int J Tuberc Lung Dis. 2013;17:432–46.

693 13. Blok L, Sahu S, Creswell J, Alba S, Stevens R, Bakker MI. Comparative meta-analysis of tuberculosis contact investigation interventions in eleven high burden countries. PLoS One. 2015;10(3):e0119822.

696 14. Creswell J, Codlin AJ, Andre E, Micek MA, Bedru A, Carter EJ, et al. Results from early programmatic implementation of Xpert MTB/RIF testing in nine countries. BMC Infect Dis. 2014a;14:2.

699 15. Creswell J, Sahu S, Blok L, Bakker MI, Stevens R, Ditiu L. A multi-site evaluation of innovative approaches to increase tuberculosis case notification: summary results. PLoS One. 2014b;9(4):9:e94465.

702 16. WHO. Implementing the End TB Strategy: The Essentials. Geneva: World Health Organization; 2015b.

704 17. Ritchie J, Lewis J. Qualitative Research Practice – A Guide for Social Science Students and Researchers. SAGE Publications; London: 2003.

706 18. Tong A, Sainsbury P, Craig J. Consolidated criteria for reporting qualitative research (COREQ): a 32-item checklist for interviews and focus groups. International Journal for Quality in Health Care. 2007;19(6):349–357.

For peer review only - http://bmjopen.bmj.com/site/about/guidelines.xhtml

19. World Bank. Classifying countries by income. Available from: https://datatopics.worldbank.org/world-development-indicators/stories/the-classification-of-countries-by-income.html. Accessed on 30 November 2019.

20. Lincoln, Guba. Lincoln YS, Guba EG. Naturalistic Inquiry. 1985; Newbury Park, CA: Sage Publications.

21. Malterud K, Siersma VD, Guassora AD. Sample size in qualitative interview studies: guided by information power. Qualitative Health Research. 2016;26(13):1753 –1760.

22. Gale N, Heath G, Cameron E, Rashid S, Redwood S. Using the framework method for the analysis of qualitative data in multi-disciplinary health research. BMC Medical Research Methodology. 2013;13:117.

23. BMJ Best Practice. What is GRADE? Available from: https://bestpractice.bmj.com/info/toolkit/learn-ebm/what-is-grade/. Accessed 24 Oct 2019.

24. University of Illinois at Chicago. What is the PICO Model. Available from: https://researchguides.uic.edu/c.php?g=252338&p=3954402. Accessed 24 Oct 2019.

25. Biermann O, Lönnroth K, Caws M, Viney K. Factors influencing active tuberculosis case finding policy development and implementation: a scoping review. BMJ Open. 2019;0:e031284. doi:10.1136/bmjopen-2019-031284.

26. Talib Y, States Burke K, Barry M. Women leaders in global health. The Lancet. 2017:5(6);PE565-566.

27. Pell C, Straus L, Andrew EV, Meñaca A, Pool R. Social and cultural factors affecting uptake of interventions for malaria in pregnancy in Africa: a systematic review of the qualitative research. PLoS One. 2011;6(7):e22452.

28. WHO. WHO handbook for guideline development. 2nd ed. Geneva: World Health Organization; 2014.

For peer review only

For peer review only - http://bmjopen.bmj.com/site/about/guidelines.xhtml

29. Daniels N. Accountability for reasonableness Establishing a fair process for priority setting is easier than agreeing on principles. BMJ. 2000;321:1300–1.

30. Karki B, Kittel G, Bolokon I, Duke T. Active community-based case finding for tuberculosis with limited resources: estimating prevalence in a remote area of Papua New Guinea. Asia Pacific Journal of Public Health. 2017;28(1):17-27.

31. Kerrigan D, West N, Tudor C, Hanrahan C, Lebina L, Msandiwa, et al. Improving active case finding for tuberculosis in South Africa: informing innovative implementation approaches in the context of the Kharitode trial through formative research. Health Research Policy and Systems. 2017;15:42.

32. Lorent N, Choun K, Malhotra S, Koeut P, Thai S, Khun KE, et al. Challenges from Tuberculosis Diagnosis to Care in Community-Based Active Case Finding among the Urban Poor in Cambodia: A Mixed-Methods Study. PLoS One. 2015;10(7):e0130179.

33. Cook VJ, Shah L, Gardy J. Modern contact investigation methods for enhancing tuberculosis control in Aboriginal communities. Int J Circumpolar Health. 2012;71:18643.

34. Adejumo AO, Azuogu B, Okorie O, Lawal OM, Onazi OJ, Gidado M, et al. Community referral for presumptive TB in Nigeria: a comparison of four models of active case finding. BMC Public Health. 2016;16:177.

35. Yassin MA, Datiko DG, Tulloch O, Markos P, Aschalew M, Shargie EB, et al. Innovative community-based approached doubled tuberculosis case notification and improve treatment outcome in Southern Ethiopia. PLoS One. 2013;8(5):e63174.).

36. Tantivess S, Tangcharoensathien V. Coverage decisions and the court: a public health perspective on glucosamine reimbursement in Thailand. Health Syst Reform. 2016;2(2):106-111. doi: 10.1080/23288604.2016.1128514.

For peer review only - http://bmjopen.bmj.com/site/about/guidelines.xhtml

37. Baltussen R, Jansen MPM, Bijlmakers L, Grutters J, Reuzel RP, Tummers M, et al. Value assessment frameworks for HTA agencies: the organization of evidence-informed deliberative processes. Value Health. 2017;20:256–60.

38. Moberg J, Oxman AD, Rosenbaum S, Schuenemann HJ, Guyatt G, Flottorp S, et al. The GRADE Evidence to Decision framework for health system and public health decisions (EtD) Health Research Policy and Systems. 2018;16:45.

39. GRADE-CERQual Project Group. Applying GRADE-CERQual to qualitative evidence synthesis findings. London: Implementation Science, BioMed Central; 2018. Available from: https://implementationscience.biomedcentral.com/articles/supplements/volume-13-supplement-1. Accessed 24 Oct 2019.

40. Lewin S, Glenton C, Munthe-Kaas H, Carlsen B, Colvin CJ, Gülmezoglu M, et al. Using qualitative evidence in decision making for health and social interventions: an approach to assess confidence in findings from qualitative evidence syntheses (GRADE-CERQual). PLoS Med. 2015;12(10):e1001895.

41. Rehfuess EA, Stratil JM, Scheel IB, Portela A, Norris SL, Baltussen R. The WHO-INTEGRATE evidence to decision framework version 1.0: integrating WHO norms and values and a complexity perspective. BMJ Glob Health 2019;4:e000844. doi:10.1136/ bmjgh-2018-000844.

42. Atun R, de Jongh T, Secci F, Ohiri K, Adeyi O. Integration of targeted health interventions into health systems: a conceptual framework for analysis. Health Policy and Planning. 2010;25:104–111. doi:10.1093/heapol/czp055.

43. Travis P, Bennett S, Haines A, Pang T, Bhutta Z, Hyder AA, Pielmeier NR, Mills A, Evans T. Overcoming health-systems constraints to achieve the Millenium Development Goals. The Lancet 2004;364:900–06.

For peer review only - http://bmjopen.bmj.com/site/about/guidelines.xhtml

44. Golub JE, Mohan CI, Comstock GW, Chaisson RE. Active case finding of tuberculosis: historical perspective and future prospects. Int J Tuberc Lung Dis. 2005;9(11):1183-1203.

45. O'Hara NN, Roy L, O'Hara LM, Spiegel JM, Lynd LD, FitzGerald JM, et al. Healthcare Worker Preferences for Active Tuberculosis Case Finding Programs in South Africa: A Best-Worst Scaling Choice Experiment. PLoS One. 2015;10(7):e0133304.

46. Lorent N, Choun K, Thai S, Kim T, Huy S, Pe R, et al. Community-Based Active Tuberculosis Case Finding in Poor Urban Settlements of Phnom Penh, Cambodia: A Feasible and Effective Strategy. PLoS One. 2014;9(3):e92754.

47. Ospina JE, Orcau A, Millet J-P, Sánchez F, Casals M, Caylà JA. Community health workers improve contact tracing among immigrants with tuberculosis in Barcelona. BMC Public Health. 2012;12:158.

48. Akkerman OW, de Lange WCM, Schölvinck EH, Wolters B, Aartsma Y, van der Werf TS, et al. Implementing tuberculosis entry screening for asylum seekers: the Groningen experience. Eur Respir J. 2016;48:261-264.

49. Datta B, Hazarika A, Shewade HD, Ayyagari K, Kumar AMV. Digital chest X-ray through a mobile van: public private partnership to detect sputum negative pulmonary TB. BMC Res Notes. 2017;10:96.

50. Corbett EL, MacPherson. Tuberculosis screening in high human immunodeficiency virus prevalence settings: turning promise into reality. Int J Tuberc Lung Dis. 2013;17(9):1125-1138.

51. Seedat F, Hargreaves S, Friedland JS. Engaging New Migrants in Infectious Disease Screening: A Qualitative Semi-Structured Interview Study ofUK Migrant Community Health-Care Leads. PLoS One. 2014;9(10):e108261.

For peer review only - http://bmjopen.bmj.com/site/about/guidelines.xhtml

52. Murphy K, Fafard P. Taking power, politics, and policy problems seriously. J Urban Health. 2012;89:4:723–732. doi: 10.1007/s11524-012-9694-6.

List of abbreviations

ACF – Active Case-Finding

COREQ – COnsolidated criteria for REporting Qualitative research

NGO – Non-governmental organization

NTP – National TB Programme

TB – Tuberculosis

WHO – World Health Organization

Ethics approval and consent to participate

This study has been approved by Regionala Etikprövningsnämnden i Stockholm (diarienummer: 2017/2281-31/2). Participants received background information about the study and provided written informed consent.

Consent for publication

Participants provided written informed consent.

For peer review only - http://bmjopen.bmj.com/site/about/guidelines.xhtml

Availability of data and materials

All data relevant to the study are included in the article or uploaded as supplementary information. The data generated and/or analysed in the study are not publicly available due to participant anonymity.

Additional files

Additional file 1: COREQ Checklist

Additional file 2: Interview guide

Competing interests

We have no competing interests to declare.

Funding

This work was supported by the EU-Horizon 2020-funded IMPACT-TB project (grant 733174).

Authors' contributions

OB, KL, MC and KV conceived the study. OB developed the interview guides, which KL, MC and KV provided feedback on. OB conducted all interviews, coded them and developed an analytical framework. OB revised the coding and the analytical framework based on SA and KV's input. OB charted the data into a framework matrix, which SA and KV provided

For peer review only

For peer review only - http://bmjopen.bmj.com/site/about/guidelines.xhtml

feedback on. OB interpreted the data writing memos for each study theme, and discussed these with SA, KL and KV. All authors read and approved the final manuscript.

Acknowledgements

OB, KL and MC are part funded by the EU-Horizon 2020-funded IMPACT-TB project (grant 733174). The authors thank the interviewees who generously shared their time to participate in the study. The authors also thank Jenny Siméus, writing instructor at Karolinska Institutet University Library, for her valuable feedback in writing this manuscript. Kerri Viney is supported by a Sidney Sax Early Career Fellowship from the Australian National Health and Medical Research Council, GNT1121611.

For peer review only

COREQ (COnsolidated criteria for REporting Qualitative research) Checklist

A checklist of items that should be included in reports of qualitative research. You must report the page number in your manuscript where you consider each of the items listed in this checklist. If you have not included this information, either revise your manuscript accordingly before submitting or note N/A.

Topic	Item No.	Guide Questions/Description	Reported on Page No.
**Domain 1: Research team and reflexivity**			
Personal characteristics			
Interviewer/facilitator	1	Which author/s conducted the interview or focus group?	
Credentials	2	What were the researcher's credentials? E.g. PhD, MD	
Occupation	3	What was their occupation at the time of the study?	
Gender	4	Was the researcher male or female?	
Experience and training	5	What experience or training did the researcher have?	
Relationship with participants			
Relationship established	6	Was a relationship established prior to study commencement?	
Participant knowledge of the interviewer	7	What did the participants know about the researcher? e.g. personal goals, reasons for doing the research	
Interviewer characteristics	8	What characteristics were reported about the inter viewer/facilitator? e.g. Bias, assumptions, reasons and interests in the research topic	
**Domain 2: Study design**			
Theoretical framework			
Methodological orientation and Theory	9	What methodological orientation was stated to underpin the study? e.g. grounded theory, discourse analysis, ethnography, phenomenology, content analysis	
Participant selection			
Sampling	10	How were participants selected? e.g. purposive, convenience, consecutive, snowball	
Method of approach	11	How were participants approached? e.g. face-to-face, telephone, mail, email	
Sample size	12	How many participants were in the study?	
Non-participation	13	How many people refused to participate or dropped out? Reasons?	
Setting			
Setting of data collection	14	Where was the data collected? e.g. home, clinic, workplace	
Presence of non-participants	15	Was anyone else present besides the participants and researchers?	
Description of sample	16	What are the important characteristics of the sample? e.g. demographic data, date	
Data collection			
Interview guide	17	Were questions, prompts, guides provided by the authors? Was it pilot tested?	
Repeat interviews	18	Were repeat inter views carried out? If yes, how many?	
Audio/visual recording	19	Did the research use audio or visual recording to collect the data?	
Field notes	20	Were field notes made during and/or after the inter view or focus group?	
Duration	21	What was the duration of the inter views or focus group?	
Data saturation	22	Was data saturation discussed?	
Transcripts returned	23	Were transcripts returned to participants for comment and/or	

For peer review only

Topic	Item No.	Guide Questions/Description	Reported on Page No.
		correction?	
**Domain 3: analysis and findings**			
Data analysis			
Number of data coders	24	How many data coders coded the data?	
Description of the coding tree	25	Did authors provide a description of the coding tree?	
Derivation of themes	26	Were themes identified in advance or derived from the data?	
Software	27	What software, if applicable, was used to manage the data?	
Participant checking	28	Did participants provide feedback on the findings?	
Reporting			
Quotations presented	29	Were participant quotations presented to illustrate the themes/findings? Was each quotation identified? e.g. participant number	
Data and findings consistent	30	Was there consistency between the data presented and the findings?	
Clarity of major themes	31	Were major themes clearly presented in the findings?	
Clarity of minor themes	32	Is there a description of diverse cases or discussion of minor themes?	

Developed from: Tong A, Sainsbury P, Craig J. Consolidated criteria for reporting qualitative research (COREQ): a 32-item checklist for interviews and focus groups. *International Journal for Quality in Health Care*. 2007. Volume 19, Number 6: pp. 349 – 357

Once you have completed this checklist, please save a copy and upload it as part of your submission. DO NOT include this checklist as part of the main manuscript document. It must be uploaded as a separate file.

For peer review only

Additional file 2. Interview guide

Basic information and active case-finding (ACF) policy and implementation work in your organization

1. What is your role in ACF policy development and/or implementation?
    - Since when have you been working in this role?
2. Could you briefly describe (current) ACF policy development and implementation supported by your organization?
    - What is the status of implementation and scale-up?
    - What do monitoring and evaluation efforts look like?
        i. Which indicators are being used?
        ii. Is there any data available/published?
    - What are the future plans for ACF in this context?

Personal view/values and preferences

3. What are the benefits of ACF in your view?
    - … at the level of the individual?
    - … at the community level?
    - … at the level of the health system?
    - … compared to other interventions for early case detection?
    - Have you/has your organization deprioritized other activities in order to prioritize resources for ACF?
        i. If so, why do you/does your organization prioritize ACF?
    - What other activities for early case detection are you/is your organization engaging in? (e.g. improve lab/diagnostic services, train health workers in identifying people with possible tuberculosis (TB), reduce financial/access barriers, address social protection)
    - Do you think the scale-up of ACF is essential for reaching the goals outlined in the End TB Strategy?
        i. If so, what's the most important action to achieve this scale-up?
        ii. Should anything else instead be prioritized less?
4. What are the risks of ACF in your opinion? (e.g. harm for individual, stigma and discrimination, cost, operational risks)
    - … at the level of the individual?
    - … at the community level?
    - … at the level of the health system?
    - … compared to other interventions for early case detection?
5. What do you think about the World Health Organization (WHO) guidelines related to ACF? (e.g. on improving early TB detection, screening guideline, screening operational guide, contact investigation)
    - Have you used any of the WHO guidelines?
    - Do you think others are using them?
    - Does anything need to be improved in these guidelines?

For peer review only - http://bmjopen.bmj.com/site/about/guidelines.xhtml

Engagement in ACF project, policy or scale up in specific country(ies)

6. Are you engaged in an ACF project in a particular country?
   - How are you engaged?
   - In which country are you engaged?
   - Could you describe an example(s)? (e.g. setting, risk group, activities, timeline)
7. Has this ACF project developed into/influenced policy?
   - If yes, what kind of policy? (e.g. become part of National Strategic Plan, sectoral policy or law)
   - If not, why?
8. Could you describe the/a ACF policy? (e.g. setting, risk group, activities, timeline)
9. Who was/is responsible for the different parts of the ACF policy cycle (agenda setting, policy formulation, implementation and evaluation)?
   - … in terms of human resources?
   - … in terms of collaboration?
   - … when it comes to technical input?
   - … when it comes to management?
10. Who was/is funding which part of the ACF policy cycle?
11. Which factors influence(d) the ACF policy development? / Which factors influence(d) the ACF policy implementation?
    - … thinking about the overall country context?
      i. How did it influence the ACF policy cycle?
    - … in terms of the health system and policy context?
      i. How did it influence the ACF policy cycle?
    - … when it comes to financing?
      i. How did it influence the ACF policy cycle?
    - Which factor is most powerful in influencing these processes?
12. Which other organizations/partners influence/influenced the ACF policy development? / Which other organizations/partners influence/influenced the ACF policy implementation?
    - … Donors (e.g. Global Fund, bilateral donors, researchers, etc.)
      i. How did they influence the ACF policy cycle?
    - … Funding mechanisms, specific funds, existing resources
      i. How did they influence the ACF policy cycle?
    - … International technical agencies (WHO, Stop TB Partnership, etc.)
      i. How did they influence the ACF policy cycle?
    - … Other organizations (civil society organization, non-governmental organizations, etc.)
      i. How did they influence the ACF policy cycle?
    - Which organization/partner is most powerful in influencing these processes?
13. When it comes to the use of evidence in the ACF policy development process… / When it comes to the use of evidence in the ACF policy implementation process…

For peer review only - http://bmjopen.bmj.com/site/about/guidelines.xhtml

- … Which type of evidence was/is being used? (e.g. global, local, scientific, tacit)?
- … What type of outcomes did/does the evidence focus on? (e.g. case detection, reduce delays, treatment outcomes, cost-effectiveness)
- … In which part of the policy cycle was/is evidence used (e.g. priority-setting, policy formulation, policy implementation, policy evaluation)?
- … What are the opportunities in using evidence for improving ACF policy cycle?
- … What are the challenges in using evidence for improving the ACF policy cycle?

14. What is your most important lesson learned related to ACF?
15. What would you like to say about future ACF policy?
16. Do you have any additional comments?

For peer review only

For peer review only - http://bmjopen.bmj.com/site/about/guidelines.xhtml

BMJ Open

"Power plays plus push": Experts' insights into the development and implementation of active tuberculosis case-finding policies globally, a qualitative study

Journal:	*BMJ Open*
Manuscript ID	bmjopen-2019-036285.R1
Article Type:	Original research
Date Submitted by the Author:	12-Mar-2020
Complete List of Authors:	Biermann, Olivia; Karolinska Institutet, Public Health Sciences<br>Atkins, Salla; Tampere University, New Social Research and Faculty of Social Sciences; Karolinska Institute, Department of Public Health Sciences<br>Lönnroth, Knut; Karolinska Institutet Department of Public Health Sciences,<br>Caws, Maxine; Liverpool School of Tropical Medicine, Department of Clinical Sciences<br>Viney, Kerri ; Karolinska Institute, Public Health Sciences
**Primary Subject Heading**:	Health policy
Secondary Subject Heading:	Global health, Infectious diseases, Public health, Qualitative research
Keywords:	PUBLIC HEALTH, Tuberculosis < INFECTIOUS DISEASES, QUALITATIVE RESEARCH, Health policy < HEALTH SERVICES ADMINISTRATION & MANAGEMENT

SCHOLARONE™
Manuscripts

I, the Submitting Author has the right to grant and does grant on behalf of all authors of the Work (as defined in the below author licence), an exclusive licence and/or a non-exclusive licence for contributions from authors who are: i) UK Crown employees; ii) where BMJ has agreed a CC-BY licence shall apply, and/or iii) in accordance with the terms applicable for US Federal Government officers or employees acting as part of their official duties; on a worldwide, perpetual, irrevocable, royalty-free basis to BMJ Publishing Group Ltd ("BMJ") its licensees and where the relevant Journal is co-owned by BMJ to the co-owners of the Journal, to publish the Work in this journal and any other BMJ products and to exploit all rights, as set out in our licence.

The Submitting Author accepts and understands that any supply made under these terms is made by BMJ to the Submitting Author unless you are acting as an employee on behalf of your employer or a postgraduate student of an affiliated institution which is paying any applicable article publishing charge ("APC") for Open Access articles. Where the Submitting Author wishes to make the Work available on an Open Access basis (and intends to pay the relevant APC), the terms of reuse of such Open Access shall be governed by a Creative Commons licence – details of these licences and which Creative Commons licence will apply to this Work are set out in our licence referred to above.

Other than as permitted in any relevant BMJ Author's Self Archiving Policies, I confirm this Work has not been accepted for publication elsewhere, is not being considered for publication elsewhere and does not duplicate material already published. I confirm all authors consent to publication of this Work and authorise the granting of this licence.

For peer review only

"Power plays plus push": Experts' insights into the development and implementation of active tuberculosis case-finding policies globally, a qualitative study

Olivia Biermann[1]* (olivia.biermann@ki.se)

Salla Atkins[1 2] (salla.atkins@tuni.fi)

Knut Lönnroth[1] (knut.lonnroth@ki.se)

Maxine Caws[3 4] (mcaws@hotmail.com)

Kerri Viney[1 5] (kerri.viney@anu.edu.au)

1 Department of Global Public Health, Karolinska Institutet, Tomtebodavägen 18a, 17177 Stockholm, Sweden

2 New Social Research and Global Health and Development, Faculty of Social Sciences, Tampere University, Kalevantie 4, 33100 Tampere, Finland

3 Department of Clinical Sciences, Liverpool School of Tropical Medicine, Pembroke Pl, Liverpool L3 5QA, United Kingdom

4 Birat Nepal Medical Trust, Lazimpat, Kathmandu, Nepal

5 Research School of Population Health, College of Health and Medicine, Australian National University, Canberra ACT 2600, Australia

*Corresponding author

For peer review only - http://bmjopen.bmj.com/site/about/guidelines.xhtml

Abstract (292 words)

Objective

To explore experts' views on factors influencing national and global active case-finding (ACF) policy development and implementation, and the use of evidence in these processes.

Design

This is an exploratory study based on semi-structured expert interviews.

Participants

The study involved a purposive sample of 39 experts from international, non-governmental and non-profit organizations, funders, government institutions, international societies, think tanks, universities and research institutions worldwide. Framework analysis was applied.

Results

This study highlighted the perceived need among experts for different types of evidence for ACF policy development and implementation, and for stakeholder engagement including researchers and policymakers to foster evidence use. Interviewees stressed the influence of government, donor and non-governmental stakeholders in ACF policy development. Such key stakeholders also influence ACF policy implementation, in addition to available systems and processes in a given health system, and implementers' motivation and incentives. According to the interviewees, the World Health Organization (WHO) guidelines for systematic screening face the innate challenge of providing guidance to countries across the broad area of ACF in terms of target groups, settings and screening algorithms. The guidelines could be improved by focusing on what *should* be done rather than what *can* be done in ACF, and by

For peer review only - http://bmjopen.bmj.com/site/about/guidelines.xhtml

For peer review only

providing *how to* examples. Leadership, integration into health systems and long-term financing are key for ACF to be sustainable.

Conclusions

We provide new insights into ACF policy processes globally, particularly regarding facilitators for and barriers to ACF policy development, evidence need and use, and donor organizations' influence. According to expert participants, national and global ACF policy development and implementation can be improved by broadening stakeholder engagement. Meanwhile, using diverse evidence to inform ACF policy development and implementation could mitigate the "power plays plus push" that might otherwise disrupt and mislead these policy processes.

Key words

Tuberculosis, active case-finding, community, policy development, policy implementation, evidence use

Article summary

Strengths and limitations of this study

- Expert interviews were able to elicit a unique insight into ACF policy development and implementation.
- Expert interviews filled knowledge gaps regarding factors influencing ACF policy development, donors' influence and evidence use in ACF policy processes.

For peer review only - http://bmjopen.bmj.com/site/about/guidelines.xhtml

- The number and diverse range of experts involved increase the study's trustworthiness and confirmability.
- Women and interviewees from low- and middle-income countries were underrepresented in the study, potentially limiting the transferability of the results.
- We did not systematically conduct analyses by stakeholder group but described the patterns we observed and highlighted the affiliations of interviewees quoted.

For peer review only

Manuscript (6,704 words)

Background

Tuberculosis (TB) is a major global health emergency, especially in low- and middle-income countries. TB is curable and preventable. Still, it remains the leading cause of death from a single infectious agent and one of the top 10 causes of death worldwide [1]. In 2019, the estimated incident TB cases and those notified globally resulted in a difference of three million cases, reflecting a combination of underreporting of detected TB cases and underdiagnosis, specifically in countries with major financial and geographic barriers to accessing care [1]. Many people with TB are diagnosed only after long delays [2-4], causing increased morbidity, much suffering and economic hardship, and sustaining transmission [1].

The World Health Organization (WHO) End TB Strategy [5] was endorsed by member states at the World Health Assembly in 2014, while the United Nations Sustainable Development Goals [6] were adopted in 2015. Both are aimed at ending the global TB epidemic. Subsequently, there has been increasing international attention on TB. In 2017, the Global Ministerial Conference on Ending TB in the Sustainable Development Era took place in Russia, with the aim of accelerating implementation of the End TB Strategy [7]. In 2018, the

For peer review only - http://bmjopen.bmj.com/site/about/guidelines.xhtml

UN held the first-ever General Assembly high-level meeting on TB in New York, which endorsed a political declaration to speed up progress towards ending TB. This declaration was adopted by the General Assembly on 10 October 2018 [8]. Both the Global Ministerial Conference and the General Assembly re-emphasized the importance of active case-finding (ACF).

Ending TB will require intensified activity to increase TB case detection [5]. One strategy for increased TB case detection is systematic screening, which is defined by the WHO as the "systematic identification of people with suspected active TB, in a predetermined target group, using tests, examinations or other procedures that can be applied rapidly" [9]. ACF is synonymous with systematic screening for active TB, although it usually implies screening outside of health facilities. ACF is mostly provider-initiated. It may target people who do not seek appropriate health care because they: a) do not have or recognize symptoms, b) do not perceive that they have a health problem requiring medical attention, or c) face barriers in accessing appropriate care [9].

ACF has been implemented for decades primarily in high-income countries, starting with mass screening campaigns in the general population in the 1950s and 1960s, then moving towards specific risk populations in recent decades, such as migrants from high-incidence countries and prison populations [10,11]. In low- and middle-income countries, the interest in ACF has increased in recent years, mainly as a response to a sustained case detection gap documented in TB prevalence surveys, annual Global TB Reports produced by WHO [1] and the development of new WHO guidelines on systematic screening [9].

Questions remain about both *if* ACF in general is worthwhile, as well as *how* to best develop and implement ACF in a given context as a synergistic, rather than parallel structure to the given health system. The evidence base is weak concerning the benefits and cost-effectiveness of ACF on both individual and community levels and how these vary between target risk

For peer review only - http://bmjopen.bmj.com/site/about/guidelines.xhtml

For peer review only

groups [12]. However, potential benefits of ACF for patients include reduced morbidity, mortality and socioeconomic consequences due to earlier diagnosis, while society can benefit from TB infection prevention, reduced transmission and a reduced burden of TB [9]. There is some evidence that TB screening in high-risk groups can significantly increase TB case notifications [13-15]. However, from the health system perspective screening can be costly and lead to diversion of scarce resources. It can also cause harm to patients, e.g. by increasing the risk of false positive diagnoses, creating an additional financial burden associated with attending screening and follow-up, or increased stigma and discrimination, if not properly targeted and implemented [16].

The potential benefits and challenges of ACF need to be carefully balanced when designing and implementing ACF. Given the relatively weak evidence base for ACF, related policy development and implementation processes rely on stakeholders' tacit knowledge, values and preferences. Yet, little is known about the latter, which potentially impact the development and implementation of national and global ACF policies. The aim of this study was to explore the views of experts on the factors that influence ACF policy development and implementation, and their views of the use of evidence in these processes.

Methods

This was an exploratory study based on semi-structured expert interviews [17]. The research team used the COREQ (COnsolidated criteria for REporting Qualitative research) Checklist [18] to report the study (*Additional file 1*).

OB is a doctoral student in public health sciences focusing on ACF with experience in qualitative research. The multidisciplinary research team consisting of a medical doctor, an

For peer review only - http://bmjopen.bmj.com/site/about/guidelines.xhtml

epidemiologist, a microbiologist and a social scientist were involved in this study to ensure different viewpoints were included on ACF policy development and implementation.

Recruitment and sample selection

The interviewees were purposively sampled to include stakeholders involved in ACF policy development and implementation based at international (n=16), non-governmental (n=2) and non-profit organizations (n=2), funders (n=4), government institutions (n=2), international societies (such as the International Society of Travel Medicine, but in the TB field) (n=2), think tanks (n=1), universities (n=6) and research institutions (n=3), as well as one independent consultant. The research team compiled the initial list of interviewees based on knowledge of networks of experts and on the published scientific literature. The list was discussed with, expanded and verified by two independent experts in the field.

The primary investigator (OB) contacted 50 individuals via email. Of these, two suggested that their colleagues be interviewed instead, eight did not reply and one declined participation due to lack of time and interest. Seven of the 11 people (64%) who declined participation were female. Table 1 provides an overview of the 39 participants who agreed to participate, their sex, professional affiliation and country where they are currently based, classified according to the World Bank [19]. In the results section, we have used quotes from interviewees across all country income levels to increase the dependability of the results [20]. Moreover, where possible in the results, we have tried to reflect all participants' voices.

Table 1. Participants and their background information (in chronological order)

ID	Sex	Affiliation	Country classification according to the World Bank [19]

For peer review only - http://bmjopen.bmj.com/site/about/guidelines.xhtml

1	M	University	High-income country
2	M	International organization	Low-income country
3	M	Government institution	Low-income country
4	M	International organization	Low-income country
5	M	Government institution	Low-income country
6	M	International organization	Low-income country
7	M	Non-governmental organization	Low-income country
8	M	Non-governmental organization	Low-income country
9	F	Research institution	High-income country
10	M	International organization	High-income country
11	M	International organization	High-income country
12	M	Research institution	High-income country
13	F	Non-profit organization	Upper middle-income country
14	F	International society	Lower middle-income country
15	M	Funder	High-income country
16	M	International organization	High-income country
17	F	International organization	High-income country
18	M	Research institution	High-income country
19	M	International organization	High-income country
20	M	University	High-income country
21	M	University	High-income country
22	M	International society	High-income country
23	F	Think tank	High-income country
24	F	International organization	High-income country

25	M	International organization	High-income country
26	M	International organization	High-income country
27	M	Independent consultant	Lower middle-income country
28	M	International organization	High-income country
29	M	International organization	Lower middle-income country
30	M	Funder	High-income country
31	M	Funder	Lower middle-income country
32	M	University	High-income country
33	M	Funder	High-income country
34	M	International organization	Lower middle-income country
35	M	International organization	High-income country
36	F	University	Low-income country
37	M	University	High-income country
38	M	Non-profit organization	High-income country
39	M	International organization	Upper middle-income country

Data collection

OB collected the data between February and May 2018 through semi-structured interviews via the phone or in person. She developed the interview guides (*Additional file 2*) which MC, KL and KV provided feedback on. The first interview was conducted as a pilot interview after which the guide was revised by making it shorter to focus on the principal topics of interest. After providing information about the study and obtaining informed written consent, OB asked the interviewees about their experience in developing and/or implementing ACF policies, factors that influenced these policy processes, and the use of evidence. The interviews were audio-recorded. No repeat interviews were carried out and no formal field

For peer review only - http://bmjopen.bmj.com/site/about/guidelines.xhtml

notes were taken. OB conducted interviews aiming to ensure that the sample would hold adequate information power to develop new knowledge [21]. The large number of participants was deemed necessary given the broad aim of the study and that all interviewees had extremely relevant experience related to different aspects of ACF policy development and implementation. This allowed capturing opinions from the diverse range of experts involved in ACF policy development and implementation, but also led to the decision to present parts of the results (on the perceived benefits and risks of ACF) in a separate article in order to do justice to the breadth and depth of the findings.

Eleven interviews were carried out in person; out of these, eight interviews were conducted during a field visit to Nepal, two during WHO meetings and one at an international organization. During the interviews, only OB and the respective interviewee were present. The typical duration of an interview was 30-60 minutes. OB transcribed 10 of the audio-recorded interviews verbatim, while the remaining ones were transcribed by a professional company The anonymity and confidentiality of the participants were ensured by unique assigned number codes and removing all identifiers except the respondent affiliation in the presentation of the results. OB offered all participants the opportunity to view their transcripts for comments or correction, however, only three participants requested to see the transcripts. No comments or corrections were made by those who chose to view the transcripts.

Data analysis

OB analysed the qualitative data from the expert interviews with *NVivo 11* using framework analysis [17]. The data were analysed abductively; identifying themes a priori, while allowing for additional themes to emerge from the data. Using the framework analysis approach as described by Gale et al. [22], OB coded all interviews and developed an analytical framework. SA and KV provided comments on the coding, based on which OB revised the codes. The

For peer review only - http://bmjopen.bmj.com/site/about/guidelines.xhtml

data was then charted into a framework matrix, on which SA and KV provided feedback. OB interpreted the data by writing memos for each study theme, and discussed these with SA, KL and KV. Table 2 provides an example of the coding process.

Table 2. Example of the coding process

**Interviewee**	**Quote**	**Code**	**Category**	**Theme**
I-27, independent consultant in a lower middle-income country	*"So, I think it's the political push that then forces the technocrats to develop policies."*	Government influencing	Government leadership and commitment	Factors influencing ACF policy development

Patient and public involvement

The preliminary findings were shared at three different scientific conferences in 2018. The interaction with participants of these events provided unique opportunities for validating the findings. For the presentation of preliminary findings at the World Union Conference on Lung Health, personalized invitations were sent to all 39 interviewees. A few interviewees attended and two provided feedback. As such, the presentation of preliminary findings gave an opportunity for member-checking. No direct changes were made based on the validation and member-checking, but these processes helped to more critically reflect on the findings. Once published, the results of this study will be reported back to each interviewee individually. In addition, targeted issue briefs will be developed for researchers and decision-makers in the field. We will also share the results with the public via a video and short messages on social media.

For peer review only - http://bmjopen.bmj.com/site/about/guidelines.xhtml

Results

We generated the following themes from the data: 1) evidence generation and use, 2) factors influencing ACF policy development, 3) factors influencing ACF policy implementation, 4) WHO guidelines on systematic screening and 5) sustainability of ACF. Table 3 provides an overview of the five main themes and the 16 related codes. The benefits and risks of ACF were additional major themes which will be analysed and discussed in a separate publication. Overall, the interviewees had a wide variety of views on ACF; from ACF being a *"waste basket"* for resources to it being *"common sense"*.

Table 3 Summary of major themes and categories related to ACF policy development and implementation

1	Evidence generation and use	1	Dissemination and exchange of evidence
		2	Demand for evidence by decision-makers
		3	Stakeholder engagement to facilitate evidence use
2	Factors influencing ACF policy development	1	Government leadership and commitment
		2	Donor funding
		3	Non-governmental organizations' experience
3	Factors influencing ACF policy implementation	1	Human and financial resources
		2	Systems, processes and resources to build on
		3	Donor funding and related target-setting
		4	Government power
		5	Health workers' motivation and incentives
4	WHO guidelines on systematic	1	Positive and negative perceptions

For peer review only - http://bmjopen.bmj.com/site/about/guidelines.xhtml

	screening	2	Contextualization of global guidelines locally
		3	Suggested improvements
5	Sustainability of ACF	1	Opportunities for sustainability
		2	Challenges for sustainability

Theme one: Evidence generation and use

Most interviewees described the evidence on ACF as being relatively limited and emphasized the need to generate different types of evidence to inform ACF policy development and implementation. They stressed the importance of disseminating and exchanging evidence, of the demand for evidence by decision-makers and stakeholder engagement to enable evidence use. Apart from highlighting specific types of evidence, interviewees across the different settings had similar views with regards to this theme.

Interviewees highlighted that a variety of evidence is needed and demanded by decision-makers working on ACF; from effectiveness and health economic evaluations to implementation and operational research. One interviewee from a university in a high-income country stressed that to demonstrate effectiveness, there is a need *"to do ACF in the context of randomized controlled trials"* (I-32). Another interviewee from a non-governmental organization (NGO) in a low-income country highlighted the importance of distinguishing clearly where the decisions are being made; be it at the community, district or national level:

> *"I think this is very important, i.e. what types of evidence you would need to make decisions at various levels (…). What evidence is enough evidence at what level to take the decision"* (I-7).

Local evidence was said by many to play a significant role in for instance available health and diagnostic facilities, and health workers' capacity and experience in communicating with communities. In particular, evidence from national TB prevalence surveys was described as

For peer review only - http://bmjopen.bmj.com/site/about/guidelines.xhtml

significant for TB policy development more broadly. Two interviewees from funding organizations in high-income countries concluded that countries should be encouraged *“to adopt [ACF] policies based on the local evidence and then move forward, rather than waiting for systematic reviews”* (I-30) and *“you should implement enough to figure out what’s practicable and what works, and then that should become policy”* (I-15).

According to the interviewees, evidence use in ACF policy development and implementation necessitates evidence dissemination and exchange, especially to share unpublished findings. One interviewee from an international organization highlighted:

> *“Unfortunately, we are [from a low-income country] and we are not very good at publishing. We’ve got a wealth of experience that is unpublished (...) but it has been presented at several conferences.”* (I-35).

Depending on the country context, gaps may exist between evidence and policy and/or between policy and practice. As one interviewee from an international organization in a high-income country pointed out:

> “*Countries are different. As I said, in [that country] (...) from evidence to policy was difficult. But once it [ACF] was inside the policy or even without the policy, they used to easily convert it to practice. But here [in our country] (...) evidence to policy is easier, but policy to practice is more difficult.”* (I-28)

Interviewees emphasized that researchers should engage with key stakeholders from the beginning of the research process to foster research use in ACF policy development and implementation; stakeholders may include the WHO, the Ministry of Health and the National TB Programme.

> *“Make sure that you have the right partners from the beginning; partners who are going to take your results and actually do something with them. Because*

For peer review only - http://bmjopen.bmj.com/site/about/guidelines.xhtml

otherwise you are kind of doing it [research on ACF] in a vacuum", said one interviewee from a university in a high-income country (I-37).

Moreover, to spark dialogue through stakeholder engagement, an interviewee from an NGO in a low-income country stressed that one must *"create platforms, or you need to use the platforms which are already there."* (I-7) Regular review meetings at sub-national and national levels to discuss challenges and successes related to ACF offer one such platform. Overall, evidence use was said to be influenced by *who* is being engaged and by personal contacts which may be *"more important than they should be",* as another interviewee from a university in a low-income country described (I-36).

Theme two: Factors influencing ACF policy development

According to the interviewees, many different stakeholders influence ACF policy development, specifically governments, donors and NGOs. Interviewees underlined stakeholder involvement as being necessary for policy development and the contextualization of global policy into local realities. Interviewees did not have any contradicting views with regards to this theme, but rather highlighted the specific roles of certain stakeholders they thought were most influential in ACF policy development.

The leadership, buy-in and commitment of governments and National TB Programmes, were described as being vital for ACF policy development and implementation. India was mentioned as a prime example where political push *"forced the technocrats to develop policies and implement them"* (I-27, an independent consultant in a lower middle-income country). Governments make decisions for political reasons or donor incentives, even if these contradict the evidence. One representative from an international organization in a high-income country highlighted an example of action perceived to be contradicting their view of the evidence:

For peer review only - http://bmjopen.bmj.com/site/about/guidelines.xhtml

> "*Women and children of reproductive age (…) should only be included as part of the passive system not as a priority for ACF ever. But when you talk to NTP [National TB Programme] managers, there is strong political pressure and a perception that donors want them to focus on women and children." (I-24)*

Donor organizations such as the Global Fund and the case-finding initiative TB REACH (the latter is coordinated by the Stop TB Partnership and funded largely by Global Affairs Canada) were described as being influential in ACF policy development, e.g. TB REACH was said to have *"brought this concept of ACF to the country"* (I-2, interviewee from an international organization in a low-income country), while the Global Fund *"hold[s] every power to change things and not to change things"* regarding ACF policy development (I-7, representative from an NGO in a low-income country). Likewise, interviewees pointed out that donors' influence was linked to WHO's influence, as donors request countries to adopt WHO guidelines to be eligible for funding:

> "*Why national policymakers are looking mainly at things like WHO documents: because a lot of them get Global Fund money and Global Fund money is often aligned with countries implementing WHO policies",* described an interviewee who is based at a research institution in a high-income country (I-9).

This observation was shared by another interviewee, from an NGO in low-income country (I-8). Linking back to the preceding theme on evidence generation and use, it seemed from the interviews that it was important to acknowledge that TB REACH projects have the potential to generate useful evidence for future policy and practice, as an independent consultant in a lower middle-income country pointed out (I-27).

Interviewees said that NGOs are often the "implementers" of ACF whose years of experience are of great value for ACF policy development and they should therefore be involved in the

For peer review only

For peer review only - http://bmjopen.bmj.com/site/about/guidelines.xhtml

same, e.g. in policy dialogues with the government and other key stakeholders. One representative of an NGO in a low-income country stated:

> "*We [NGOs] are the one who really deal with the people (…). We have the evidence. We have the good photographs. We have the data. (…) We are the ones who can influence [ACF policy development].*" (I-8)

Theme 3: Factors influencing ACF policy implementation

Interviewees elaborated on available resources, systems, processes and resources within a given health system, donor and government stakeholders, as well as the motivation and incentives for health workers as major factors influencing ACF policy implementation. Interviewees emphasized the role of particular stakeholders, as well as barriers and facilitators they thought were most influential in ACF policy implementation, while no clearly contradictory views on this theme emerged.

The implementation and scale-up of ACF policies depends on the availability of financial resources, as many interviewees stressed.

> *"We realized that in a country like [our country], we have great policies. The problem is the implementation. (…) And this is where the support of the development partners, funded through PEPFAR, have been key to implement these policies, particularly ACF policies",* one interviewee described (I-39, interviewee from an international organization in an upper middle-income country).

ACF implementation may *"just stop because [there is] no funding"* (I-29, interviewee from an international organization in a lower middle-income country). An interviewee from a funding organization in a high-income country provided a different perspective regarding the funding for ACF by highlighting that *"ACF through government funding can be more*

For peer review only - http://bmjopen.bmj.com/site/about/guidelines.xhtml

difficult than doing it through donor funding" (I-15). This perspective may inhibit long-term thinking about ACF, as it seems to focus on immediate action to implement rather than sustainability, which is more likely to come with government investment. In addition to limited financial resources, human resource constraints for ACF were highlighted as a major challenge by experts from low-, middle- and high-income countries. These constraints could hinder National TB Programmes in thinking more strategically and ambitiously about how to address TB comprehensively.

The use of existing systems and processes in a given health system was said to be central because *"if you start from scratch, it [ACF] is much more difficult than if there are already things to which you can link"*, as an interviewee from an international organization in a high-income country pointed out (I-17). Interviewees mentioned that ACF policy implementation can build upon experience from existing screening programmes (e.g. cervical cancer screening), activities for vulnerable populations (e.g. needle exchange programmes), health care infrastructure (e.g. chest X-ray buses), already known locations for screening in high incidence areas and trained human resources (e.g. those involved in prevalence surveys). Yet, pursuing synergies may be challenging due to the fragmentation of activities. In addition, the structure and financing of TB within a health system matters in terms of availability of resources:

> *"TB has tended to fall into the preventative [arm of the health system] and that has limited the availability for resources",* described an interviewee from a university in a high-income country (I-32).

Processes including supportive supervision, monitoring and the use of Standard Operating Procedures are critical for ACF policy implementation and are necessary to avoid corruption, interviewees discussed. In one country, the *"whole case-finding system collapsed along with the supervision"* (I-16, interviewee from an international organization in a high-income

For peer review only - http://bmjopen.bmj.com/site/about/guidelines.xhtml

country). Moreover, processes that strengthen communication with, engagement of and awareness-raising among communities were described as instrumental for ACF policy implementation, e.g. to help reduce stigma. One interviewee from a university in a high-income country mentioned how the community *"has started to advocate loudly for ACF services"* (I-20).

Many interviewees underlined that donors influence the implementation of ACF policies in countries with no or insufficient domestic resources. *"The piper will determine what music you play"* (I-35, interviewee from an international organization in a high-income country), which, again, highlights the power which donor organizations are perceived to have in influencing ACF policy implementation, and the possible resulting lack of a sense of policy ownership in some countries. Donors influence ACF policy implementation by setting targets for their funding recipients and pushing them towards reaching them. One interviewee from an international organization in a high-income country described:

> *"These targets that countries have set, that donors have set; people are very anxious (...) and that often means the easiest short cut is to do ACF, even if it is a little bit unethical or little bit using low specificity tools, so you have a little bit of over diagnosis. Donors are very comfortable with that."* (I-24)

The consequences of implementing ACF under donor pressure are unclear and should be balanced against the unethical nature of inaction on the TB epidemic, but scale-up of inaccurate diagnostic strategies might lead to heightening the potential risks of ACF such as increasing false-positive diagnoses, as the interviewee mentioned.

ACF policy development and implementation depend on *"power plays plus push"*, e.g. in a country with no written ACF policy, ACF was still being implemented because the National TB Programme manager was respected and able to push for it (I-29, interviewee from an international organization in a lower middle-income country). The aforementioned pressure

For peer review only

403 by politicians, donors and WHO may be seen as additional examples of "power plays plus
404 push". Many interviewees highlighted the important role of power dynamics in ACF policy
405 implementation. It seems crucial to be aware of such dynamics, while the use of evidence may
406 help mitigate them. ACF policy implementation is in itself a balancing act, which power
407 imbalances might negatively impact.

408 The motivation of health workers and volunteers is an important enabler for ACF policy
409 implementation. These "implementers" can be strongly motivated by their desire to help
410 people, by understanding the benefit of ACF for communities, by receiving feedback on the
411 outcomes of their work (e.g. using performance indicators) and/or by feeling ownership of the
412 ACF process, according to the interviewees. Financial and non-financial incentives (e.g.
413 salaries, transportation allowances, provision of motorbikes or mobile airtime) have a
414 significant role in motivating health workers and volunteers to implement ACF as an outreach
415 activity, interviewees discussed. Nevertheless, incentives can raise expectations and distort
416 ACF policy implementation in the long-term, e.g. if government health workers are paid extra
417 as part of an ACF project, they will also expect an extra pay for such activities in the future
418 and for other work; another balancing act. While incentives should be in line with what a
419 country could adopt later, they are often difficult or impossible for governments to sustain, an
420 interviewee said.

421

422 **Theme 4: WHO guidelines on systematic screening**

423 This theme focuses on stakeholders' perceptions of the WHO guidelines on systematic
424 screening, the need for their contextualization and suggestions for improving them. This
425 theme elicited different views among stakeholders, which are described in the following.

426 The WHO guidelines on systematic screening are perceived positively by many, e.g. as a
427 reference document when planning ACF activities as well as to put ACF on the agenda.

For peer review only - http://bmjopen.bmj.com/site/about/guidelines.xhtml

Positive perceptions of the guidelines were described by interviewees from different countries, while negative perceptions were only voiced by interviewees in high-income countries. Such negative perceptions included the guidelines being vague, lacking information about the *how-to* of ACF and being unduly negative in terms of mentioning the risk of increasing false-positive diagnoses through ACF. Low-income countries may be more receptive to and reliant on WHO guidelines, while in a middle-income country *"you've got really serious domestic universities providing the formal policy evidence. And the country kind of says 'Thanks but no thanks' to outside opinions. They are really driving their own decisions. WHO is really not consulted very much, if at all",* a representative of a funding organization in a high-income country described (I-15). An interviewee from an international organization in a high-income country said:

> *"When you have something that is so broad – and you're talking about ACF which can be so many different things – it's just very hard to have something that works the same way in different countries (…). I think that's the main shortcoming around the guidance."* (I-28)

Interviewees emphasized the necessity of contextualizing the WHO guidelines on systematic screening, e.g. depending on a country's income level, epidemiology and availability of diagnostic tools. One interviewee from a funding organization in a high-income country pointed out that *"you just can't be as prescriptive and exact as you are in the more clinical guidelines*" (I-15), which seems like an important observation and reminder about the limitations that ACF policies will always have. According to the interviewees, contextualization of guidelines can happen in a stepwise approach, e.g. a country pilots the use of a guideline before adopting and adapting it.

Review meetings with WHO and other partners can provide a platform for discussions around guideline adaptation, interviewees said. Yet, countries have faced challenges in

For peer review only - http://bmjopen.bmj.com/site/about/guidelines.xhtml

contextualization, e.g. WHO recommends using chest X-ray which was too expensive in a country and could thus not be used (I-4, from an international organization in a low-income country). In another instance, WHO describes how contacts of an index TB patient should provide their address, while individuals were hesitant to do so due to the stigma surrounding TB in the country (I-14, interviewee from an international society in a lower middle-income country). More support for the contextualization of guidelines may be needed.

The interviewees suggested that the WHO guidelines for systematic screening [9] must be updated based on new evidence, e.g. evidence from prevalence surveys, gender analyses, studies about specific risk groups (e.g. drug users and indigenous populations) and what works and how this works, with regards to ACF. In this process, WHO should be aware of and avoid conflicts of interest, e.g. by ensuring potential conflicts of interests are adequately declared and managed. This comment is in line with what an interviewee previously highlighted about the role of personal contacts to bridge the research-policy gap. These types of biases may undermine the integrity of the process and the resulting quality of guidelines and policies.

Some interviewees lamented that WHO can be paralyzed by the need to use the strongest evidence available and suggested that the organization should consider more programmatic, less scientifically rigorous data. One interviewee from a university in a high-income country described:

> *"Usually we're relying very heavily on WHO for global policy using the GRADE[1] approach with the PICO[2] and all that stuff. I think that's laudable, but*

---

[1] GRADE (Grading of Recommendations Assessment, Development, and Evaluation) was developed for creating summaries of research evidence to help guide health decision-making. It is currently the most widely used tool for evaluating the quality of science, with more than 110 organizations endorsing the method [23].

[2] The PICO acronym stands for: P – Patient, Problem or Population; I – Intervention; C – Comparison, control or comparator; O – Outcome(s) (e.g. pain, fatigue, nausea, infections, death). The PICO process (or framework) is a mnemonic used in evidence-based practice to frame and answer a clinical or health care related question. The PICO framework is also used to develop literature search strategies, e.g. in systematic reviews [24].

For peer review only - http://bmjopen.bmj.com/site/about/guidelines.xhtml

> *sometimes I find that weird, subjected to the tyranny of the great process, and you don't make progress in smaller areas with a paucity of evidence."* (I-21)

In addition, interviewees pointed out that WHO recommendations should be based on what *should* be done, not on what *can* be done. For example, countries (not WHO) have to be the ones to decide about their ability to pay for Xpert MTB/RIF® as a diagnostic tool. This point of view illustrates a stark contrast to the contextualization challenges mentioned above, e.g. where the use of X-ray was recommended, but, frustratingly, was unable to be applied in a country as it was not feasible to implement. Moreover, the WHO guidelines could be improved by not only describing the *what,* but the *how* of systematic screening including ACF, many interviewees said. One interviewee from an NGO in a low-income country suggested:

> *"You can come up with different scenarios: 'If the context is this, then...', 'If the context is that, then…'. (...) Unless guidelines presents [the] 'how' better, (...) it's meaningless."* (I-7)

Theme 5: Sustainability of ACF

The sustainability of ACF was a cross-cutting theme in this analysis. Interviewees elaborated on opportunities and challenges related to sustainability.

> *"TB is not a like smallpox or polio. It's a long-term sustainable (...) matter",* an independent consultant in a lower middle-income country described (I-27).

That is, even more perseverance and long-term thinking may be required to end TB. Interviewees expressed similar views and concerns regarding this theme.

Interviewees highlighted that the interest in and leadership for ACF through the government

For peer review only - http://bmjopen.bmj.com/site/about/guidelines.xhtml

and the National TB Programme are important for the sustainability of ACF. Additionally, the sustainability of ACF requires its integration in and funding through the given health system. An interviewee from an international organization in a low-income country described:

> *"If this [ACF] were to be sustainable, it should start with the initiation of the NTP [National TB Programme]. (...) It has to be supported, facilitated, monitored. Because it is actually the NTP which later needs to uptake that." (I-2)*

Many interviewees highlighted the important role of National TB Programmes.

The sustainability of ACF may be restricted in places with frequent government and staff turnover, which makes it difficult to get long-term commitment for ACF from decision-makers, interviewees stressed. Of course, such turnover will affect areas beyond ACF. Also, ACF cannot be sustainable, if it depends on donor funding. One interviewee from an international organization in a high-income country summarized the situation as follows:

> *"It [ACF] is difficult to sustain. Most of the activities that have been done for ACF have been project-based. (...) So, the Global Fund comes and says: 'Here is a pot of money for ACF for the next three years.' (...) And then USAID comes (...). Or TB REACH (...). And people do it. But that's not a sustainable way of doing this and this should be part and parcel of routine programming."* (I-35)

Discussion

In summary, this study accentuated experts' perceived need for different types of evidence for ACF policy development and implementation, and for stakeholder engagement to foster evidence use. Interviewees stressed the influence of government, donor and NGO

For peer review only - http://bmjopen.bmj.com/site/about/guidelines.xhtml

stakeholders as influential players in ACF policy development. Such key stakeholders also influence ACF policy implementation, in addition to available systems and processes in a given health system and implementers' motivation and incentives. The WHO guidelines for systematic screening were said to face the innate challenge of covering the broad area of ACF in terms of target groups, settings and screening algorithms. Interviewees suggested that the guidelines could be improved by incorporating new and different types of evidence, by focusing on what *should* be done rather than what *can* be done, and by providing examples of the *how* of ACF. Finally, for ACF to be sustainable, interviewees stressed the need for leadership for ACF, its integration into health systems and the transition from donor to government funding.

Building on a broad evidence base

Interviewees emphasized the need for a variety of evidence, such as impact and economic evaluations, operational and qualitative research. Qualitative evidence has proven essential in developing and implementing health policies including in low- and middle-income countries, e.g. to prevent and treat malaria during pregnancy [25]. In the case of ACF, decision-makers may need qualitative evidence on, for example, factors influencing participation in ACF or the retention of health workers. Likewise, qualitative evidence syntheses have emerged as an important approach to inform national and global health policy development and implementation [26] and could also be useful for improving future ACF policies.

Making and implementing better ACF policies through stakeholder engagement

Successful ACF policy development and implementation necessitate stakeholder engagement, interviewees highlighted. Stakeholder engagement is an inclusive process essential for achieving legitimate decisions, which are accepted by the population and conducive to

For peer review only

effective implementation [27]. Specifically, interviewees stressed the importance of community engagement to enhance the implementation of ACF. Available evidence also shows the importance of community engagement and support for ACF implementation, e.g. through collaboration with respected community leaders (i.e. chiefs, civic leaders, village elders and counsellors) [28,29]. In addition, familiarity with the community [30] and community buy-in [31] as well as community appreciation and respect through the engagement of community health workers were said to be important [32,33]. Stakeholder engagement is also relevant for the development of WHO guidelines at the global level, and their adaptation to the national or subnational levels, where a wide array of stakeholders with diverse sets of values should be involved [34,35].

Moving from "paralyzing" to "empowering" WHO guidance

The interviewees had many suggestions for improving the WHO guidelines on systematic screening [9], questioning the appropriateness of only using the GRADE approach in the context of ACF. The WHO guidelines make graded recommendations about screening specific risk groups for TB, including three strong recommendations[3] and four conditional recommendations[4] [9]. The conditionality makes decision-making in ACF complex by leaving recommendations open to interpretation. For example, the conditionality may "paralyze" decision-makers to move screening outside of health facilities, as ACF in many vulnerable groups is only conditionally recommended. However, despite conditional recommendations and "low-quality" or "very low-quality evidence" that all of the WHO's recommendations on systematic screening are based on [9], decision-makers must still act, either in deciding to

[3] Strong recommendations: Screening in household contacts and other close contacts, people living with HIV and current and former workers in workplaces with silica exposure.

[4] Conditional recommendations: Screening among prisoners, in people with an untreated fibrotic lesion seen on chest X-ray, in settings where the TB prevalence in the general population is 100/100 000 population or higher, in geographically defined subpopulations with extremely high levels of undetected TB and other subpopulations that have very poor access to health care.

For peer review only - http://bmjopen.bmj.com/site/about/guidelines.xhtml

implement or taking the decision not to. The Global Fund and TB REACH can provide guidance in interpreting the guidelines. Yet, countries should guarantee that these interpretations and adaptations are based on the local epidemiology, health system capacity, resources, feasibility, effects and economic impact, etc. This would be paramount in order not to move away from the guidelines' original intention. Ensuring continuous monitoring and evaluation is therefore important [36]. GRADE-CERQual (Grading of Recommendations Assessment, Development and Evaluation-Confidence in the evidence from reviews of qualitative research) [37,38] may be a useful resource for future global systematic TB screening guideline development. It has been developed to assess confidence in findings from qualitative evidence syntheses. Additionally, the GRADE Evidence to Decision Framework for Health System and Public Health Decisions [36] or the WHO-INTEGRATE Evidence to Decision Framework [39] could be valuable to assess evidence for a complex intervention such as ACF.

Integrating ACF into health systems for sustainability

Interviewees underlined the need to integrate ACF into a given health system for it to be sustainable. Such integration may start with an assessment of the given health system context to understand available structures (e.g. infrastructure, budget structure and trained human resources) and processes (e.g. supportive supervision and monitoring). Interviewees described these resources as being paramount to link to and build upon. The fact that participants highlighted the need for health system integration, which seems to be relevant for any health intervention, may indicate that such integration cannot be taken for granted and/or might not always occur in ACF. It is important to acknowledge that "integration" may describe a variety of organizational arrangements across different settings [40]. Additionally, in many low-income countries, interventions generally operate through a complex patchwork of arrangements, rather than through totally stand-alone or totally integrated approaches [41].

For peer review only - http://bmjopen.bmj.com/site/about/guidelines.xhtml

To embed ACF into health systems, available systems for outreach and health promotion [4], laboratory networks [42] and free services [43] have been highlighted. Moreover, given the importance of community health workers for implementing ACF, their integration into the health system has been emphasized [44,45]. Importantly, the collaboration between various actors has been described as key for sustainable ACF implementation. The latter includes collaboration between public health practitioners and clinicians [46], district TB teams and government health staff [47], health care staff and community health workers [30,44]. Moreover, collaboration between HIV and TB sectors [48], with laboratory staff [44] and with community organizations [48,49] has been described as important. Government, National TB Programmes, WHO and donors, whose key roles in ACF policy development and implementation have been described by the interviewees, should contribute to the long-term thinking and long-term action related to ACF and towards ending TB. Murphy and colleagues [50] emphasize that only a mix of appropriate evidence, key stakeholders, processes and structures would be a solution for evidence-informed policy development and implementation.

Future research

Implementation research that sheds light on what works for whom and under which conditions may be particularly helpful to answer some of the *how* questions which our study exposed. Moreover, operational research that uses available local data, e.g. on TB notifications, may help inform local decision-making around ACF. Finally, mixed-methods studies can help explore the complexity of ACF policy development and implementation in the future, as they have the potential to both increase contextual understanding and reduce biases.

For peer review only - http://bmjopen.bmj.com/site/about/guidelines.xhtml

Strengths and limitations

While the available evidence in this area often focuses on ACF policy implementation [51], this study fills important knowledge gaps by identifying factors influencing ACF policy development and characterizing evidence use in ACF policy development and implementation, from the perspective of experts in the field. Moreover, this study offers an increased understanding of donor organizations' influence on ACF policy processes. The number and diverse range of experts involved in this study, as well as the member-checking carried out, increase the study's trustworthiness, including its confirmability and transferability [52]. The transferability of this study's results may be limited given that only a minority of the experts were from low- and middle-income countries (38%; 15 out of 39 experts). Nevertheless, all had working experience from low-and middle-income countries. Seven of the interviews with experts from low- and middle-income countries were conducted with experts from Nepal. Though all of them have different affiliations, their perspectives may be overrepresented. The results may furthermore be limited as an even smaller minority were women (18%; 7 out of 39 experts). The gender bias reflects the lack of gender parity in leadership positions in the field of global health [26]. We did not systematically conduct analyses by stakeholder group but described the patterns we observed and highlighted the affiliations of interviewees quoted..

Conclusion

Based on a variety of experts' perspectives, we generated new insights on ACF policy processes, in particular regarding facilitators for and barriers to ACF policy development, evidence need and use, and donor organizations' influence. Still, we know little about *how* to strengthen those facilitators, *how* to overcome those barriers and *how* to strengthen research use. Bringing together these different views creates a more comprehensive picture of ACF

For peer review only

For peer review only - http://bmjopen.bmj.com/site/about/guidelines.xhtml

policy development and implementation today and indicates ways to strengthen such processes in the future: National and global ACF policy development and implementation can be improved by broadening stakeholder engagement and ownership; from decision-makers at the Ministry of Health to community leaders and members. Meanwhile, using diverse evidence to inform ACF policy development and implementation could mitigate the "power plays plus push" that might otherwise disrupt and mislead these policy processes. Our findings complement the existing evidence base and can inform future national and global ACF policy processes.

References

1. WHO. Global TB Report 2019. Geneva: World Health Organization; 2019.
2. Storla DG, Yimer S, Bjune GA. A systematic review of delay in the diagnosis and treatment of tuberculosis. BMC Public Health. 2008;8(15).
3. Corbett EL, Bandason T, Duong T, Dauya E, Makamure B, Churchyard GJ, Williams BG, et al. Comparison of two active case-finding strategies for community-based diagnosis of symptomatic smear-positive tuberculosis and control of infectious tuberculosis in Harare, Zimbabwe (DETECTB): a cluster-randomised trial. Lancet 2010;376:1244-53.
4. Lönnroth K, Corbett E, Golub J, Godfrey-Faussett P, Uplekar M, Weil D, et al. Systematic screening for active tuberculosis: rationale, definitions and key considerations. Int J Tuberc Lung Dis. 2013;17(3):289-298.
5. WHO. The End TB Strategy. Global strategy and targets for tuberculosis prevention, care and control after 2015. Geneva: World Health Organization; 2015a.
6. UN. Transforming our world: The 2030 agenda for sustainable development. USA, New York: United Nations; 2015.

For peer review only - http://bmjopen.bmj.com/site/about/guidelines.xhtml

7. WHO. Ministerial Conference on TB, 2017. Available from: https://www.who.int/mediacentre/events/2017/ministerial-conference-tb/en/. Accessed on 22 October 2019.

8. UN. Political declaration of the high-level meeting of the General Assembly on the fight against tuberculosis. USA, New York: United Nations; 2018.

9. WHO. Systematic screening for active tuberculosis: principles and recommendations. Geneva: World Health Organization; 2013.

10. Eang MT, Satha P, Yadav RP, Morishita F, Nishikiori N, van-Maaren P, et al. Early detection of tuberculosis through community-based active case finding in Cambodia. BMC Public Health. 2012;12:469. doi: 10.1186/1471-2458-12-469.

11. Tyler PJ. 'The Celluloid Strip' – Mass screening for Tuberculosis in New South Wales, 1950-1975. Journal & Proceedings of the Royal Society of New South Wales. 2003;136:29-43.

12. Kranzer K, Afnan-Holmes H, Tomlin K, Golub JE, Shapiro AE, Schaap A, et al. The benefits to communities and individuals of screening for active tuberculosis disease: a systematic review. Int J Tuberc Lung Dis. 2013;17:432–46.

13. Blok L, Sahu S, Creswell J, Alba S, Stevens R, Bakker MI. Comparative meta-analysis of tuberculosis contact investigation interventions in eleven high burden countries. PLoS One. 2015;10(3):e0119822.

14. Creswell J, Codlin AJ, Andre E, Micek MA, Bedru A, Carter EJ, et al. Results from early programmatic implementation of Xpert MTB/RIF testing in nine countries. BMC Infect Dis. 2014a;14:2.

15. Creswell J, Sahu S, Blok L, Bakker MI, Stevens R, Ditiu L. A multi-site evaluation of innovative approaches to increase tuberculosis case notification: summary results. PLoS One. 2014b;9(4):9:e94465.

For peer review only - http://bmjopen.bmj.com/site/about/guidelines.xhtml

16. WHO. Implementing the End TB Strategy: The Essentials. Geneva: World Health Organization; 2015b.

17. Ritchie J, Lewis J. Qualitative Research Practice – A Guide for Social Science Students and Researchers. SAGE Publications; London: 2003.

18. Tong A, Sainsbury P, Craig J. Consolidated criteria for reporting qualitative research (COREQ): a 32-item checklist for interviews and focus groups. International Journal for Quality in Health Care. 2007;19(6):349–357.

19. World Bank. Classifying countries by income. Available from: https://datatopics.worldbank.org/world-development-indicators/stories/the-classification-of-countries-by-income.html. Accessed on 30 November 2019.

20. Lincoln, Guba. Lincoln YS, Guba EG. Naturalistic Inquiry. 1985; Newbury Park, CA: Sage Publications.

21. Malterud K, Siersma VD, Guassora AD. Sample size in qualitative interview studies: guided by information power. Qualitative Health Research. 2016;26(13):1753 –1760.

22. Gale N, Heath G, Cameron E, Rashid S, Redwood S. Using the framework method for the analysis of qualitative data in multi-disciplinary health research. BMC Medical Research Methodology. 2013;13:117.

23. BMJ Best Practice. What is GRADE? Available from: https://bestpractice.bmj.com/info/toolkit/learn-ebm/what-is-grade/. Accessed 24 Oct 2019.

24. University of Illinois at Chicago. What is the PICO Model. Available from: https://researchguides.uic.edu/c.php?g=252338&p=3954402. Accessed 24 Oct 2019.

25. Pell C, Straus L, Andrew EV, Meñaca A, Pool R. Social and cultural factors affecting uptake of interventions for malaria in pregnancy in Africa: a systematic review of the qualitative research. PLoS One. 2011;6(7):e22452.

For peer review only - http://bmjopen.bmj.com/site/about/guidelines.xhtml

26. WHO. WHO handbook for guideline development. 2nd ed. Geneva: World Health Organization; 2014.

27. Daniels N. Accountability for reasonableness Establishing a fair process for priority setting is easier than agreeing on principles. BMJ. 2000;321:1300–1.

28. Karki B, Kittel G, Bolokon I, Duke T. Active community-based case finding for tuberculosis with limited resources: estimating prevalence in a remote area of Papua New Guinea. Asia Pacific Journal of Public Health. 2017;28(1):17-27.

29. Kerrigan D, West N, Tudor C, Hanrahan C, Lebina L, Msandiwa, et al. Improving active case finding for tuberculosis in South Africa: informing innovative implementation approaches in the context of the Kharitode trial through formative research. Health Research Policy and Systems. 2017;15:42.

30. Lorent N, Choun K, Malhotra S, Koeut P, Thai S, Khun KE, et al. Challenges from Tuberculosis Diagnosis to Care in Community-Based Active Case Finding among the Urban Poor in Cambodia: A Mixed-Methods Study. PLoS One. 2015;10(7):e0130179.

31. Cook VJ, Shah L, Gardy J. Modern contact investigation methods for enhancing tuberculosis control in Aboriginal communities. Int J Circumpolar Health. 2012;71:18643.

32. Adejumo AO, Azuogu B, Okorie O, Lawal OM, Onazi OJ, Gidado M, et al. Community referral for presumptive TB in Nigeria: a comparison of four models of active case finding. BMC Public Health. 2016;16:177.

33. Yassin MA, Datiko DG, Tulloch O, Markos P, Aschalew M, Shargie EB, et al. Innovative community-based approached doubled tuberculosis case notification and improve treatment outcome in Southern Ethiopia. PLoS One. 2013;8(5):e63174.).

For peer review only

34. Tantivess S, Tangcharoensathien V. Coverage decisions and the court: a public health perspective on glucosamine reimbursement in Thailand. Health Syst Reform. 2016;2(2):106-111. doi: 10.1080/23288604.2016.1128514.

35. Baltussen R, Jansen MPM, Bijlmakers L, Grutters J, Reuzel RP, Tummers M, et al. Value assessment frameworks for HTA agencies: the organization of evidence-informed deliberative processes. Value Health. 2017;20:256–60.

36. Moberg J, Oxman AD, Rosenbaum S, Schuenemann HJ, Guyatt G, Flottorp S, et al. The GRADE Evidence to Decision framework for health system and public health decisions (EtD) Health Research Policy and Systems. 2018;16:45.

37. GRADE-CERQual Project Group. Applying GRADE-CERQual to qualitative evidence synthesis findings. London: Implementation Science, BioMed Central; 2018. Available from: https://implementationscience.biomedcentral.com/articles/supplements/volume-13-supplement-1. Accessed 24 Oct 2019.

38. Lewin S, Glenton C, Munthe-Kaas H, Carlsen B, Colvin CJ, Gülmezoglu M, et al. Using qualitative evidence in decision making for health and social interventions: an approach to assess confidence in findings from qualitative evidence syntheses (GRADE-CERQual). PLoS Med. 2015;12(10):e1001895.

39. Rehfuess EA, Stratil JM, Scheel IB, Portela A, Norris SL, Baltussen R. The WHO-INTEGRATE evidence to decision framework version 1.0: integrating WHO norms and values and a complexity perspective. BMJ Glob Health 2019;4:e000844. doi:10.1136/ bmjgh-2018-000844.

40. Atun R, de Jongh T, Secci F, Ohiri K, Adeyi O. Integration of targeted health interventions into health systems: a conceptual framework for analysis. Health Policy and Planning. 2010;25:104–111. doi:10.1093/heapol/czp055.

For peer review only - http://bmjopen.bmj.com/site/about/guidelines.xhtml

764 41. Travis P, Bennett S, Haines A, Pang T, Bhutta Z, Hyder AA, Pielmeier NR, Mills A,
765 Evans T. Overcoming health-systems constraints to achieve the Millenium
766 Development Goals. The Lancet 2004;364:900–06.

767 42. Golub JE, Mohan CI, Comstock GW, Chaisson RE. Active case finding of
768 tuberculosis: historical perspective and future prospects. Int J Tuberc Lung Dis.
769 2005;9(11):1183-1203.

770 43. O'Hara NN, Roy L, O'Hara LM, Spiegel JM, Lynd LD, FitzGerald JM, et al.
771 Healthcare Worker Preferences for Active Tuberculosis Case Finding Programs in
772 South Africa: A Best-Worst Scaling Choice Experiment. PLoS One.
773 2015;10(7):e0133304.

774 44. Lorent N, Choun K, Thai S, Kim T, Huy S, Pe R, et al. Community-Based Active
775 Tuberculosis Case Finding in Poor Urban Settlements of Phnom Penh, Cambodia: A
776 Feasible and Effective Strategy. PLoS One. 2014;9(3):e92754.

777 45. Ospina JE, Orcau A, Millet J-P, Sánchez F, Casals M, Caylà JA. Community health
778 workers improve contact tracing among immigrants with tuberculosis in Barcelona.
779 BMC Public Health. 2012;12:158.

780 46. Akkerman OW, de Lange WCM, Schölvinck EH, Wolters B, Aartsma Y, van der
781 Werf TS, et al. Implementing tuberculosis entry screening for asylum seekers: the
782 Groningen experience. Eur Respir J. 2016;48:261-264.

783 47. Datta B, Hazarika A, Shewade HD, Ayyagari K, Kumar AMV. Digital chest X-ray
784 through a mobile van: public private partnership to detect sputum negative pulmonary
785 TB. BMC Res Notes. 2017;10:96.

786 48. Corbett EL, MacPherson. Tuberculosis screening in high human immunodeficiency
787 virus prevalence settings: turning promise into reality. Int J Tuberc Lung Dis.
788 2013;17(9):1125-1138.

For peer review only

49. Seedat F, Hargreaves S, Friedland JS. Engaging New Migrants in Infectious Disease Screening: A Qualitative Semi-Structured Interview Study ofUK Migrant Community Health-Care Leads. PLoS One. 2014;9(10):e108261.

50. Murphy K, Fafard P. Taking power, politics, and policy problems seriously. J Urban Health. 2012;89:4:723–732. doi: 10.1007/s11524-012-9694-6.

51. Biermann O, Lönnroth K, Caws M, Viney K. Factors influencing active tuberculosis case finding policy development and implementation: a scoping review. BMJ Open. 2019;0:e031284. doi:10.1136/bmjopen-2019-031284.

52. Talib Y, States Burke K, Barry M. Women leaders in global health. The Lancet. 2017:5(6);PE565-566.

List of abbreviations

ACF – Active Case-Finding

COREQ – COnsolidated criteria for REporting Qualitative research

NGO – Non-governmental organization

TB – Tuberculosis

WHO – World Health Organization

Ethics approval and consent to participate

This study has been approved by Regionala Etikprövningsnämnden i Stockholm (diarienummer: 2017/2281-31/2). Participants received background information about the study and provided written informed consent.

For peer review only

Consent for publication

Participants provided written informed consent.

Availability of data and materials

All data relevant to the study are included in the article or uploaded as supplementary information. The data generated and/or analysed in the study are not publicly available due to participant anonymity.

Additional files

Additional file 1: COREQ Checklist

Additional file 2: Interview guide

Competing interests

We have no competing interests to declare.

Funding

This work was supported by the EU-Horizon 2020-funded IMPACT-TB project (grant 733174).

Authors' contributions

For peer review only

OB, KL, MC and KV conceived the study. OB developed the interview guides, which KL, MC and KV provided feedback on. OB conducted all interviews, coded them and developed an analytical framework. OB revised the coding and the analytical framework based on SA and KV's input. OB charted the data into a framework matrix, which SA and KV provided feedback on. OB interpreted the data writing memos for each study theme, and discussed these with SA, KL and KV. All authors read and approved the final manuscript.

Acknowledgements

OB, KL and MC are part funded by the EU-Horizon 2020-funded IMPACT-TB project (grant 733174). The authors thank the interviewees who generously shared their time to participate in the study. The authors also thank Jenny Siméus, writing instructor at Karolinska Institutet University Library, for her valuable feedback in writing this manuscript. Kerri Viney is supported by a Sidney Sax Early Career Fellowship from the Australian National Health and Medical Research Council, GNT1121611.

For peer review only

COREQ (COnsolidated criteria for REporting Qualitative research) Checklist

A checklist of items that should be included in reports of qualitative research. You must report the page number in your manuscript where you consider each of the items listed in this checklist. If you have not included this information, either revise your manuscript accordingly before submitting or note N/A.

Topic	Item No.	Guide Questions/Description	Reported on Page No.
**Domain 1: Research team and reflexivity**			
Personal characteristics			
Interviewer/facilitator	1	Which author/s conducted the interview or focus group?	
Credentials	2	What were the researcher's credentials? E.g. PhD, MD	
Occupation	3	What was their occupation at the time of the study?	
Gender	4	Was the researcher male or female?	
Experience and training	5	What experience or training did the researcher have?	
Relationship with participants			
Relationship established	6	Was a relationship established prior to study commencement?	
Participant knowledge of the interviewer	7	What did the participants know about the researcher? e.g. personal goals, reasons for doing the research	
Interviewer characteristics	8	What characteristics were reported about the inter viewer/facilitator? e.g. Bias, assumptions, reasons and interests in the research topic	
**Domain 2: Study design**			
Theoretical framework			
Methodological orientation and Theory	9	What methodological orientation was stated to underpin the study? e.g. grounded theory, discourse analysis, ethnography, phenomenology, content analysis	
Participant selection			
Sampling	10	How were participants selected? e.g. purposive, convenience, consecutive, snowball	
Method of approach	11	How were participants approached? e.g. face-to-face, telephone, mail, email	
Sample size	12	How many participants were in the study?	
Non-participation	13	How many people refused to participate or dropped out? Reasons?	
Setting			
Setting of data collection	14	Where was the data collected? e.g. home, clinic, workplace	
Presence of non-participants	15	Was anyone else present besides the participants and researchers?	
Description of sample	16	What are the important characteristics of the sample? e.g. demographic data, date	
Data collection			
Interview guide	17	Were questions, prompts, guides provided by the authors? Was it pilot tested?	
Repeat interviews	18	Were repeat inter views carried out? If yes, how many?	
Audio/visual recording	19	Did the research use audio or visual recording to collect the data?	
Field notes	20	Were field notes made during and/or after the inter view or focus group?	
Duration	21	What was the duration of the inter views or focus group?	
Data saturation	22	Was data saturation discussed?	
Transcripts returned	23	Were transcripts returned to participants for comment and/or	

For peer review only

Topic	Item No.	Guide Questions/Description	Reported on Page No.
		correction?	
**Domain 3: analysis and findings**			
Data analysis			
Number of data coders	24	How many data coders coded the data?	
Description of the coding tree	25	Did authors provide a description of the coding tree?	
Derivation of themes	26	Were themes identified in advance or derived from the data?	
Software	27	What software, if applicable, was used to manage the data?	
Participant checking	28	Did participants provide feedback on the findings?	
Reporting			
Quotations presented	29	Were participant quotations presented to illustrate the themes/findings? Was each quotation identified? e.g. participant number	
Data and findings consistent	30	Was there consistency between the data presented and the findings?	
Clarity of major themes	31	Were major themes clearly presented in the findings?	
Clarity of minor themes	32	Is there a description of diverse cases or discussion of minor themes?	

Developed from: Tong A, Sainsbury P, Craig J. Consolidated criteria for reporting qualitative research (COREQ): a 32-item checklist for interviews and focus groups. *International Journal for Quality in Health Care*. 2007. Volume 19, Number 6: pp. 349 – 357

Once you have completed this checklist, please save a copy and upload it as part of your submission. DO NOT include this checklist as part of the main manuscript document. It must be uploaded as a separate file.

For peer review only

Additional file 2. Interview guide

Basic information and active case-finding (ACF) policy and implementation work in your organization

1. What is your role in ACF policy development and/or implementation?
    - Since when have you been working in this role?
2. Could you briefly describe (current) ACF policy development and implementation supported by your organization?
    - What is the status of implementation and scale-up?
    - What do monitoring and evaluation efforts look like?
        i. Which indicators are being used?
        ii. Is there any data available/published?
    - What are the future plans for ACF in this context?

Personal view/values and preferences

3. What are the benefits of ACF in your view?
    - … at the level of the individual?
    - … at the community level?
    - … at the level of the health system?
    - … compared to other interventions for early case detection?
    - Have you/has your organization deprioritized other activities in order to prioritize resources for ACF?
        i. If so, why do you/does your organization prioritize ACF?
    - What other activities for early case detection are you/is your organization engaging in? (e.g. improve lab/diagnostic services, train health workers in identifying people with possible tuberculosis (TB), reduce financial/access barriers, address social protection)
    - Do you think the scale-up of ACF is essential for reaching the goals outlined in the End TB Strategy?
        i. If so, what's the most important action to achieve this scale-up?
        ii. Should anything else instead be prioritized less?
4. What are the risks of ACF in your opinion? (e.g. harm for individual, stigma and discrimination, cost, operational risks)
    - … at the level of the individual?
    - … at the community level?
    - … at the level of the health system?
    - … compared to other interventions for early case detection?
5. What do you think about the World Health Organization (WHO) guidelines related to ACF? (e.g. on improving early TB detection, screening guideline, screening operational guide, contact investigation)
    - Have you used any of the WHO guidelines?
    - Do you think others are using them?
    - Does anything need to be improved in these guidelines?

For peer review only - http://bmjopen.bmj.com/site/about/guidelines.xhtml

Engagement in ACF project, policy or scale up in specific country(ies)

6. Are you engaged in an ACF project in a particular country?
    - How are you engaged?
    - In which country are you engaged?
    - Could you describe an example(s)? (e.g. setting, risk group, activities, timeline)
7. Has this ACF project developed into/influenced policy?
    - If yes, what kind of policy? (e.g. become part of National Strategic Plan, sectoral policy or law)
    - If not, why?
8. Could you describe the/a ACF policy? (e.g. setting, risk group, activities, timeline)
9. Who was/is responsible for the different parts of the ACF policy cycle (agenda setting, policy formulation, implementation and evaluation)?
    - … in terms of human resources?
    - … in terms of collaboration?
    - … when it comes to technical input?
    - … when it comes to management?
10. Who was/is funding which part of the ACF policy cycle?
11. Which factors influence(d) the ACF policy development? / Which factors influence(d) the ACF policy implementation?
    - … thinking about the overall country context?
        i. How did it influence the ACF policy cycle?
    - … in terms of the health system and policy context?
        i. How did it influence the ACF policy cycle?
    - … when it comes to financing?
        i. How did it influence the ACF policy cycle?
    - Which factor is most powerful in influencing these processes?
12. Which other organizations/partners influence/influenced the ACF policy development? / Which other organizations/partners influence/influenced the ACF policy implementation?
    - … Donors (e.g. Global Fund, bilateral donors, researchers, etc.)
        i. How did they influence the ACF policy cycle?
    - … Funding mechanisms, specific funds, existing resources
        i. How did they influence the ACF policy cycle?
    - … International technical agencies (WHO, Stop TB Partnership, etc.)
        i. How did they influence the ACF policy cycle?
    - … Other organizations (civil society organization, non-governmental organizations, etc.)
        i. How did they influence the ACF policy cycle?
    - Which organization/partner is most powerful in influencing these processes?
13. When it comes to the use of evidence in the ACF policy development process… / When it comes to the use of evidence in the ACF policy implementation process…

For peer review only - http://bmjopen.bmj.com/site/about/guidelines.xhtml

- … Which type of evidence was/is being used? (e.g. global, local, scientific, tacit)?
- … What type of outcomes did/does the evidence focus on? (e.g. case detection, reduce delays, treatment outcomes, cost-effectiveness)
- … In which part of the policy cycle was/is evidence used (e.g. priority-setting, policy formulation, policy implementation, policy evaluation)?
- … What are the opportunities in using evidence for improving ACF policy cycle?
- … What are the challenges in using evidence for improving the ACF policy cycle?

14. What is your most important lesson learned related to ACF?
15. What would you like to say about future ACF policy?
16. Do you have any additional comments?

For peer review only
